# Selection of Reference Genes for qRT-PCR Analysis in Medicinal Plant *Glycyrrhiza* under Abiotic Stresses and Hormonal Treatments

**DOI:** 10.3390/plants9111441

**Published:** 2020-10-26

**Authors:** Yuping Li, Xiaoju Liang, Xuguo Zhou, Zhigeng Wu, Ling Yuan, Ying Wang, Yongqing Li

**Affiliations:** 1 Key Laboratory of South China Agricultural Plant Molecular Analysis and Genetic Improvement & Guangdong Provincial Key Laboratory of Applied Botany, South China Botanical Garden, Chinese Academy of Sciences, Guangzhou 510650, China; liyuping222@scbg.ac.cn (Y.L.); 2190525004@fafu.edu.cn (X.L.); wuzhigeng@scbg.ac.cn (Z.W.); lyuan3@uky.edu (L.Y.); 2 Center of Economic Botany, Core Botanical Gardens, Chinese Academy of Sciences, Guangzhou 510650, China; 3 University of Chinese Academy of Sciences, Beijing 100049, China; 4 Department of Entomology, University of Kentucky, Lexington, KY 40546, USA; xuguozhou@uky.edu; 5 Department of Plant and Soil Sciences, University of Kentucky, Lexington, KY 40546, USA; 6 Gannan Normal University, Ganzhou 341000, China

**Keywords:** reference gene, abiotic stress, hormonal treatment, *Glycyrrhiza*, medicinal plant

## Abstract

Best known as licorice, *Glycyrrhiza* Linn., a genus of herbaceous perennial legume, has been used as a traditional herbal medicine in Asia and a flavoring agent for tobacco and food industry in Europe and America. Abiotic stresses and hormonal treatments can significantly impact the development and metabolism of secondary metabolites in *Glycyrrhiza*. To better understand the biosynthesis of the trace-amount bioactive compounds, we first screened for the suitable reference genes for quantitative real-time reverse transcription PCR (qRT-PCR) analysis in *Glycyrrhiza.* The expression profiles of 14 candidate reference genes, including *Actin1* (*ACT*)*, Clathrin complex AP1* (*CAC*), *Cyclophilin* (*CYP*), *Heat-shock protein 40* (*DNAJ*), *Dehydration responsive element binding gene* (*DREB*), *Translation elongation factor1* (*EF1*), *Ras related protein* (*RAN*), *Translation initiation factor* (*TIF1*), *β-Tubulin* (*TUB*), *Ubiquitin-conjugating enzyme E2* (*UBC2*), *ATP binding-box transpoter 2* (*ABCC2*), *COP9 signal compex subunit 3* (*COPS3*), *Citrate synthase* (*CS*), and *R3H domain protein 2* (*R3HDM2*) from two congeneric species, *Glycyrrhiza uralensis* F. and *Glycyrrhiza inflata* B., were examined under abiotic stresses (osmotic and salinity) and hormonal treatments (Abscisic acid (ABA) and methyl jasmonic acid (MeJA)) using a panel of software, including geNorm, NormFinder, BestKeeper, and Delta CT. The overall stability, however, was provided by RefFinder, a comprehensive ranking system integrating inputs from all four algorithms. In *G. uralensis*, the most stable reference genes under osmotic stress, salt stress, ABA treatment, and MeJA treatment were *TIF1*, *DNAJ*, *CS*, and *ABCC2* for leaves and *DNAJ*, *DREB*, *CAC*, and *CAC* for roots, respectively. In comparison, the top ranked genes were *TUB*, *CAC*, *UBC2*, and *RAN* for leaves and *TIF1*, *ABCC2*, *CAC*, and *UBC2* for roots, respectively, under stress and hormonal treatments in *G. inflata*. *ACT* and *TIF1*, on the other hand, were the least stable genes under the most experimental conditions in the two congeneric species. Finally, our survey of the reference genes in legume shows that *EF*, *ACT*, *UBC2*, and *TUB* were the top choices for the abiotic stresses while *EF*, *UBC2*, *CAC*, and *ABCC2* were recommended for the hormonal treatments in Leguminosae. Our combined results provide reliable normalizers for accurate gene quantifications in *Glycyrrhiza* species, which will allow us to exploit its medicinal potential in general and antiviral activities in particular.

## 1. Introduction

**Figure 1:**
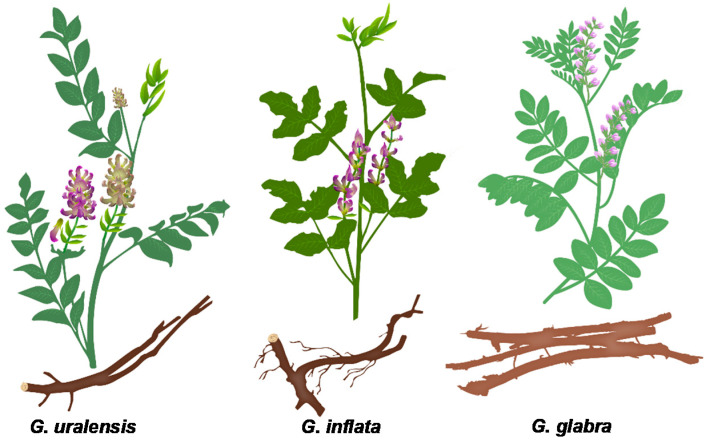
Schematic drawing of the three congeneric *Glycyrrhiza* species.

Medicinal plants have been documented as an important source for discovering new pharmaceutical molecules [1]. Best known as licorice, *Glycyrrhiza* is a genus of medicinal perennial herbs within the legume family (Fabaceae), native to Asia and the Mediterranean region and currently distributed in temperate and subtropical regions of the world [2]. Included in the Pharmacopoeia of the People’s Republic of China, three congeneric species, *Glycyrrhiza uralensis* F., *G. inflata* B., and *Glycyrrhiza glabra* L. (Figure 1), have been used extensively in antiquity and traditional herbal medicine. Glycyrrhizin, a glycoside and the major bioactive component, has showed promise against a broad spectrum of respiratory, hepatic, and systemic viral diseases. The eminent threats to public health and national security by global pandemics of viral diseases, including influenza; HIV; and recently, the two highly contagious respiratory diseases caused by coronaviruses, severe acute respiratory syndrome (SARS) and COVID-19, and the increasing issues with drug resistances (e.g., influenza and HIV) demand the development of effective antiviral alternatives with different mode of actions [3]. *Glycyrrhiza* sp.-derived bioactive compounds is a rich reservoir for developing drugs treating SARS [4,5] and COVID-19 [6]. In addition to its medicinal values, licorice extract has also been used as a flavoring agent for tobacco and food industry due to the sweet taste of glycyrrhizic acid (170 times as sweet as sucrose) [7]. Because of its utility in medical field and food industry, in conjunction with the advent of the genomics era, molecular and genomic research in *Glycyrrhiza* is the future [8,9].

Gene expression analysis provides basic information of functional genes. Quantitative real-time reverse transcription PCR (qRT-PCR) is the most common general lab technique to accurately determine gene expression levels due to its high throughput, accuracy, and sensitivity [10,11]. Stably expressed internal reference genes are critical for accurate normalization of the expression of the gene of interest [12]. Housekeeping genes, due to their indispensable function for survival, are good candidates for reference gene selection [13,14,15]. However, more and more evidences have shown that the expression levels of housekeeping genes are not always constant [16,17]. Therefore, it is essential to test whether selected candidates can be used as reference genes under the corresponding conditions [18].

Biosynthesis and accumulation of trace-amount bioactive metabolites in medicinal plants are regulated by multiple intrinsic and extrinsic factors through corresponding genes and gene networks. Glycyrrizin, the major active compound of licorice, only happens in roots and rhizomes [19]. Moreover, the underground part of licorice is also useful for human beings. Licorice shoots are a kind of high-quality forage grass because of their high content of coarse fiber and flavonoid [20], while similar flavonoids, such as isotrifoliol and glisoflavanone, reported to have anti-inflammatory effects, are only accumulated in the underground part of licorice [21]. Therefore, it is germane to carry out tissue-specific studies in *Glycyrrhiza*.

*Glycyrrhiza* mainly distributes in dry or semi-dry areas, including Eurasia, northern Africa, and western Asia. Drought and salt stresses, the most common abiotic factors in licorice habitats, have been reported to induce the accumulation of glycyrrhizin [22]. Plant hormones play a vital role in plant adaptation to the environment and can regulate plant growth, development, and nutrient allocation [23]. Abscisic acid (ABA) and methyl jasmonic acid (MeJA) are two important abiotic elicitors involved in plant responses to drought, salt or osmotic stresses [24], and secondary metabolism [25]. Although reference genes under the drought stresses in *G. glabra* [26] have been documented, information concerning suitable reference genes for *Glycyrrhiza* species is still lacking in general, especially for two other congeneric species with pharmaceutical potentials, *G. uralensis* and G. *inflata*. 

In the present research, we hypothesized that there are overlapping reference genes recommended for congeneric *Glycyrrhiza* species under different abiotic stresses or hormonal treatments. To examine this hypothesis, we (1) evaluated the stability of 14 candidate reference genes, (2) selected optimal reference genes under abiotic stresses and hormonal treatments, and (3) compared the suitable reference genes in three congeneric *Glycyrrhiza* species under the osmotic stress. In addition, we surveyed and summarized the reference genes previously used within Leguminosae plants.

## 2. Results

### 2.1. Expression Profiling of Candidate Reference Genes 

**Figure 2:**
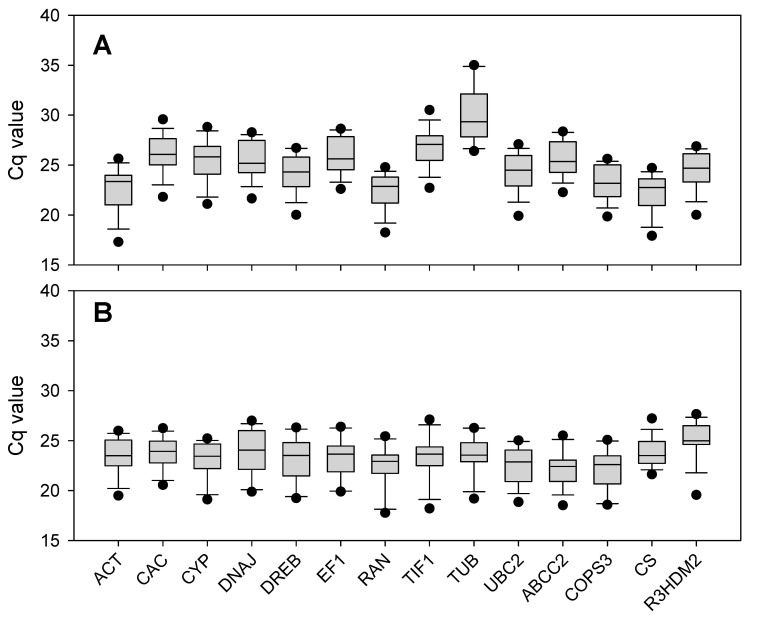
Distribution of threshold cycle (Cq) values for candidate reference genes: distribution of threshold cycle (Cq) values of (**A**) *G. uralensis* and (**B**) *G. inflata* in all samples. Boxes: the interquartile range; lines across the boxes: median; the line above and below the boxes: the maximum and minimum values; and black dots: outliers.

In this study, a total of 14 candidate genes, *including Actin1* (*ACT*), *Clathrin complex AP1* (*CAC*), *Cyclophilin* (*CYP*), *Heat-shock protein 40* (*DNAJ*), *Dehydration responsive element binding gene* (*DREB*), *Translation elongation factor1* (*EF1*), *Ras related protein* (*RAN*), *Translation initiation factor* (*TIF1*), *β-Tubulin* (*TUB*), *Ubiquitin-conjugating enzyme E2* (*UBC2*), *ATP binding-box transpoter 2* (*ABCC2*), *COP9 signal compex subunit 3* (*COPS3*), *Citrate synthase* (*CS*), and *R3H domain protein 2* (*R3HDM2*), were selected and evaluated. The expression levels of the candidate reference genes were determined as quantification cycle (Cq) values. As shown in Figure 2, *DREB* possesses the lowest gene expression variation in *G. uralensis*, with a narrow range of Cq values from 24.26 to 28.96, revealing that *DREB* might have a stable expression level under different treatments. Conversely, *TUB* has Cq values that range from 22.74 to 34.74, so *TUB* is probably not a good choice for a reference gene in *G. uralensis* (Table S1). For the *G. inflata*, *RAN* and *TIF1* presented the lowest or highest gene expression variations, respectively, with the Cq values ranging from 22.81 to 26.34 or 17.18 to 26.50 (Table S2).

For the 14 candidate genes, *TIF1* had the lowest Cq value both in *G. uralensis* (20.74) and *G. inflata* (20.17), indicating the highest expression level of *TIF1* in the two species, while *EF1* (27.31) or *CAC* (25.62) was expressed at low levels in *G. uralensis* or *G. inflata*, respectively (Figure 2 and Table S3). The transcripts of 14 candidate genes showed different levels of abundance in *G. uralensis* and *G. inflata*. The mean Cq values of the genes ranged from 19 to 27, with the majority falling between 22 and 26 across all tested samples in *G. uralensis* (Table S3), and the mean Cq values of the genes ranged from 18 to 26, with the majority falling between 22 and 25 across all tested samples in *G. inflata* (Table S3). Therefore, the expression levels of these candidate genes were much higher in tested samples in *G. inflata* than in *G. uralensis*.

### 2.2. Stability of Candidate Reference Genes

The expression profiles of the 14 candidate reference genes in *G. uralensis* and *G. inflata* roots and/or leaves across all experiments in this study were analyzed using geNorm, NormFinder, BestKeeper, and Delta CT; the stability order analyzed by each method are listed in Table S4 and Table S5.

For normal condition in *G. uralensis*, the top three most stable candidate reference genes for leaves were *DNAJ*, *DREB*, and *UBC2* identified by geNorm; *ABCC2*, *CS*, and *UBC2* identified by NormFinder; *COPS3*, *DREB*, and *CAC* identified by BestKeeper; and *CS*, *ABCC2*, and *UBC2* identified by Delta CT, while the most stable reference genes in the roots were *DNAJ*, *UBC2*, and *CAC* recommended by geNorm and NormFinder; *COPS3*, *EF1,* and *R3HDM2* recommended by BestKeeper; and *DNAJ*, *CAC*, and *UBC2* recommended by Delta CT (Table S4). In *G. inflata*, the most stable reference genes of leaves were *TUB*, *COPS3,* and *CYP* recommended by geNorm; *TUB*, *COPS3,* and *RAN* recommended by NormFinder and Delta CT; and *R3HDM2*, *CAC,* and *DNAJ* recommended by BestKeeper, while the top three most stable candidate reference genes for roots were *CS*, *CAC*, and *ABCC2* identified by geNorm; *CS*, *CAC*, and *R3HDM2* identified by NormFinder; *ACT*, *ABCC2*, and *R3HDM2* identified by BestKeeper; and *CS*, *CAC*, and *R3HDM2* identified by Delta CT (Table S5).

For the osmotic stress (treatment with 100 mM mannitol) condition, the most stable reference genes were *DNAJ*, *RAN*, and *CS* recommended by geNorm under osmotic stress in *G. uralensis* leaves; *ABCC2*, *TUB*, and *EF1* recommended by NormFinder; *COPS3*, *DREB*, and *CYP* recommended by the BestKeeper; and *DNAJ*, *TIF1*, and *R3HDM2* recommended by Delta CT. In the roots of *G. uralensis* under osmotic stress, the top three most stable candidate reference genes were *DNAJ*, *CAC*, and *DREB* identified by geNorm, NormFinder, and Delta CT and *COPS3*, *R3HDM2*, and *ABCC2* identified by BestKeeper (Table S4). In *G. inflata*, the most stable reference genes were *RAN*, *TUB,* and *CYP* recommended by geNorm; *TUB*, *DNAJ*, and *RAN* recommended by NormFinder; *DNAJ*, *COPS3,* and *R3HDM2* recommended by BestKeeper; and *COPS3*, *TUB,* and *R3HDM2* recommended by Delta CT in the leaves. The most stable reference genes were *TIF1*, *R3HDM2,* and *UBC2* recommended by geNorm; *TIF1*, *R3HDM2*, and *ABCC2* recommended by NormFinder; *CS*, *ABCC2,* and *UBC2* recommended by BestKeeper; and *TIF1*, *R3HDM2,* and *ABCC2* recommended by Delta CT in the roots (Table S5).

Under salt stress (treatment with 100 mM NaCl), the most stable reference genes were *DNAJ*, *CAC*, and *UBC2* recommended by geNorm in *G. uralensis* leaves; *ABCC2*, *CYP*, and *TIF1* recommended by NormFinder; *DREB*, *RAN*, and *CS* recommended by the BestKeeper; and *CYP*, *ABCC2*, and *DNAJ* recommended by Delta CT. In the roots of *G. uralensis* under salt stress, the top three most stable candidate reference genes were *DREB*, *UBC2*, and *DNAJ* identified by geNorm; *DNAJ*, *TUB*, and *DREB* identified by NormFinder and Delta CT; and *DREB*, *COPS3*, and *UBC2* identified by BestKeeper (Table S4). In *G. inflata*, the most stable reference genes were *RAN*, *TUB,* and *CAC* recommended by geNorm; *CAC*, *COPS3*, and *DNAJ* recommended by NormFinder; *COPS3*, *R3HDM2,* and *RAN* recommended by BestKeeper; and *CAC*, *DNAJ,* and *RAN* recommended by Delta CT in the leaves. The most stable reference genes were *COPS3*, *R3HDM2,* and *RAN* recommended by geNorm; *ABCC2*, *CS*, and *CYP* recommended by NormFinder; *DNAJ*, *ABCC2,* and *EF1* recommended by BestKeeper; and *CAC*, *ABCC2,* and *CYP* recommended by Delta CT in the roots (Table S5).

For ABA treatment (100 μM ABA), the most stable reference genes were *UBC2*, *CS,* and *CAC* recommended by geNorm under ABA treatment in *G. uralensis* leaves; *CS*, *CAC,* and *ABCC2* recommended by NormFinder and Delta CT; and *TUB*, *R3HDM2,* and *CS* recommended by BestKeeper. In the roots of *G. uralensis* under ABA treatment, the top three most stable candidate reference genes were *CAC*, *RAN*, and *UBC2* identified by geNorm, NormFinder, and Delta CT amd *COPS3*, *R3HDM2*, and *TUB* identified by BestKeeper (Table S4). In *G. inflata*, the most stable reference genes were *DREB*, *UBC2,* and *ABCC2* recommended by geNorm, *COPS3*, *CS*, and *CYP* by NormFinder; *UBC2*, *CS,* and *DREB* recommended by BestKeeper; and *CS*, *COPS3,* and *DREB* recommended by Delta CT in the leaves. The most stable reference were *DREB*, *ABCC2,* and *UBC2* genes recommended by geNorm; *CAC*, *TUB*, and *COPS3* recommended by NormFinder; *CYP*, *R3HDM2,* and *CS* recommended by BestKeeper; and *CAC*, *TUB,* and *CS* recommended by Delta CT in the roots (Table S5).

Under MeJA treatment (100 μM MeJA), the most stable reference genes were *DREB*, *UBC2*, and *DNAJ* recommended by geNorm in *G. uralensis* leaves; *ABCC2*, *CAC*, and *CYP* recommended by NormFinder; *R3HDM2*, *ABCC2*, and *EF1* recommended by BestKeeper; and *CAC*, *ABCC2*, and *UBC2* recommended by Delta CT. In the roots of *G. uralensis* under MeJA treatment, the top three most stable candidate reference genes were *ABCC2*, *CS*, and *EF1* identified by geNorm; *CAC*, *TUB*, and *UBC2* identified by NormFinder and Delta CT; and *EF1*, *COPS3*, and *R3HDM2* identified by BestKeeper (Table S4). In *G. inflata*, the most stable reference genes were *DNAJ*, *RAN,* and *CAC* recommended by geNorm; *COPS3*, *TIF1*, and *UBC2* recommended by NormFinder; *COPS3*, *R3HDM2,* and *TIF1* recommended by BestKeeper; and *UBC2*, *RAN*, and *DNAJ* recommended by Delta CT in the leaves. The most stable reference genes were *UBC2*, *ABCC2,* and *DREB* recommended by geNorm; *UBC2*, *CS*, and *DREB* recommended by NormFinder and Delta CT; and *DREB*, *COPS3,* and *ACT* recommended by BestKeeper in the roots (Table S5).

### 2.3. Optimal Reference Genes Under Different Experimental Conditions 

Based on RefFinder, a web-based software, comprehensive ranking of reference genes integrating all four software results above was done. For the normal condition in *G. uralensis*, the most stable reference gene were *CS*, *DNAJ*, and *DREB* in the leaves and *DNAJ*, *UBC2*, and *CAC* in the roots (Table 1 and Table S4). In *G. inflata*, the most stable reference gene were *COPS3*, *TUB*, and *RAN* in the leaves and *CS*, *CAC*, and *R3HDM2* in the roots (Table 1 and Table S5). For the *G. glabra* studied previously, the most stable reference genes were *UBC2*, *EF1*, and *ACT* in the leaves and *TUB*, *ACT*, and *UBC2* in the roots [26].

Under osmotic stress, *TIF1*, *DNAJ*, and *RAN* were identified as the most stable reference genes in the leaves of *G. uralensis* by RefFinder, while *DNAJ*, *CAC*, and *DREB* were that in the roots (Table 1 and Table S4). In *G. inflata*, *TUB*, *COPS3*, and *DNAJ* were the most stable reference genes in the leaves, while *TIF1*, *R3HDM2*, and *ABCC2* were that in the roots (Table 1 and Table S5). For the *G. glabra* studied previously, the most stable reference genes under drought stress were *ACT, UBC2*, and *TIF1* in the leaves and *UBC2*, *ACT*, and *TUB* in the roots [26]. For salt stress condition, *DNAJ*, *CYP*, and *CAC* were identified as the most stable reference genes in the leaves of *G. uralensis* by RefFinder, while *DREB*, *DNAJ*, and *UBC2* were that in the roots (Table 1 and Table S4). In *G. inflata*, *CAC*, *RAN*, and *COPS3* were the most stable reference genes in the leaves, while *ABCC2*, *CAC*, and *DNAJ* were that in the roots (Table 1 and Table S5).

For hormonal treatments, the most stable reference genes based on the RefFinder algorithms were *CS*, *CAC*, and *UBC2* and *UBC2*, *CS*, and *DREB* under ABA treatment in the *G. uralensis* and *G. inflata* leaves, respectively, and *CAC*, *RAN*, and *UBC2* and *CAC*, *TUB*, and *CS* under ABA treatment in the *G. uralensis* and *G. inflata* root, respectively (Table 1, Table S4 and S5). To analyze gene expression under MeJA treatment, *ABCC2*, *UBC2*, and *CAC* were the most stable reference genes according to RefFinder in the *G. uralensis* leaves and *CAC*, *TUB*, and *ABCC2* were that in the root (Table 1 and Table S4). *RAN*, *COPS3*, and *UBC2* were identified as the most stable reference genes in the *G. inflata* leaves, and *UBC2*, *DREB*, and *CS* were identified in the root (Table 1 and Table S5).

**Figure 3:**
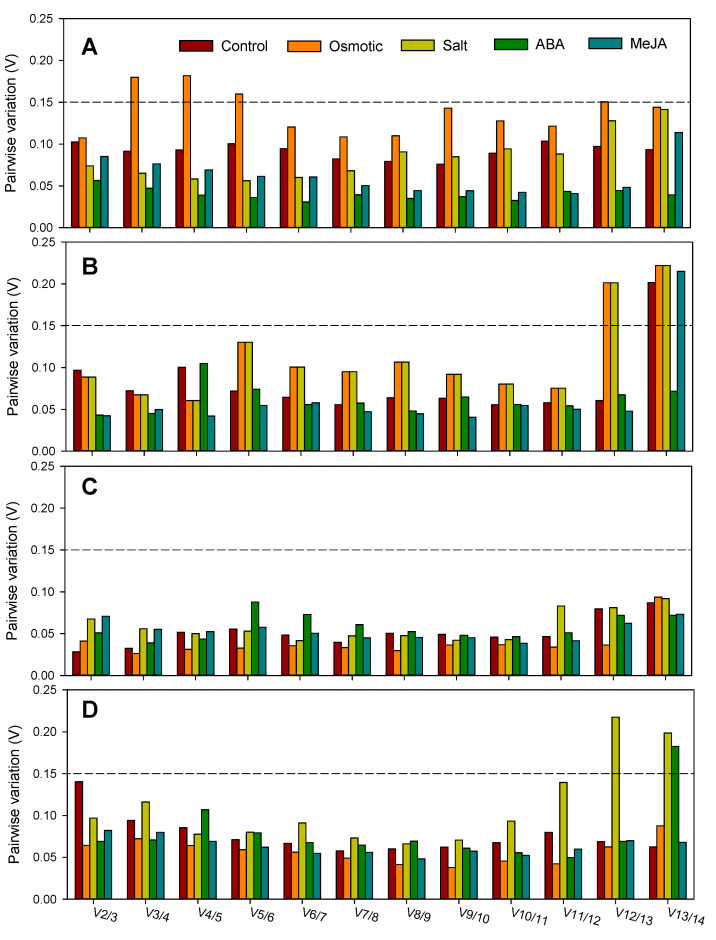
Pairwise variation analysis of the candidate reference genes of *G. uralensis* and *G. inflata*: the pairwise variation (V_n_/V_n+1_) in the leaf (**A**) or root (**B**) of *G. uralensis* and the leaf (**C**) or root (**D**) of *G. inflata*. Pairwise variation analysis was conducted by geNorm procedure. V_n_/V_n+1_ was calculated with the normalization factors NF_n_ and NF_n+1_ to determine the optimal number of reference genes required for quantitative real-time reverse transcription PCR (qRT-PCR) data normalization under different conditions.

The optimal number of reference genes under each experimental condition required for reliable normalization in two species were predicted by geNorm software with the pairwise variation (V) values (cutoff = 0.15). When pairwise variations Vn/*n*+1 < 0.15, it means that an addition reference gene (*n*+1) is not necessary. For all the experimental conditions in *G. uralensis* and *G. inflata*, the first V-value less than 0.15 occurred at V2/3 (Figure 3), suggesting that two reference genes were adequate to correctly normalize gene expression.

### 2.4. Comparison of the Suitable Reference Genes under Different Experimental Conditions

**Figure 4:**
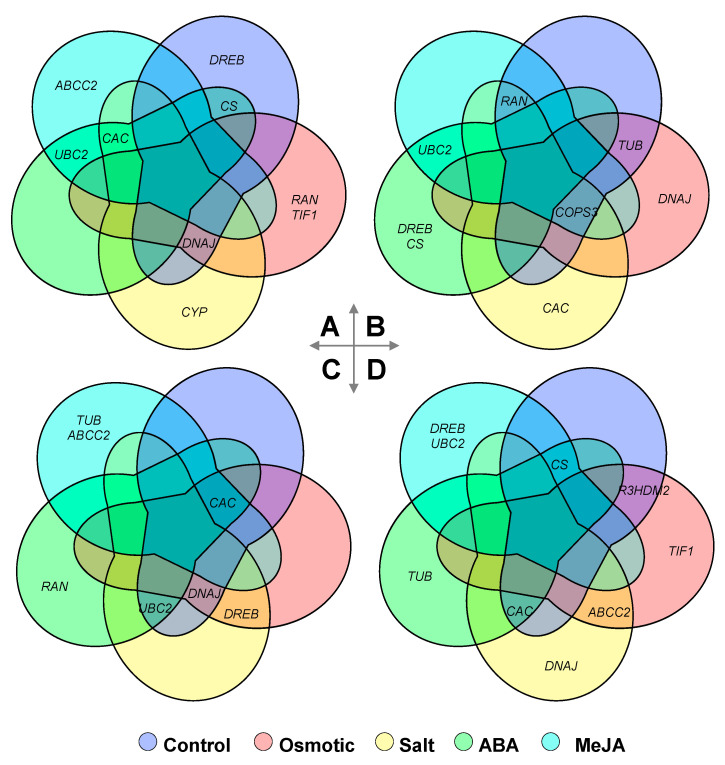
Comparison of the optimal reference genes under different experimental conditions between *G. urelensis* and *G. inflata*: the Venn diagram compares the top three most suited reference genes under different experimental conditions in the leaf (**A**,**B**) or root (**C**,**D**) between the congeneric *G. uralensis* and *G. inflate*, respectively.

In order to analyze the reference genes under different experimental conditions systematically, we compared the suitable reference genes for different experimental condition tested in this study. Results showed that the optimal reference genes for the same tissues of certain *Glycyrrhiza* species are different under various experimental conditions and that no one of the top three candidates was shared among all experimental conditions tested (Figure 4). A total of ten genes appeared in the top three list in *G. uralensis*, among which *CAC*, *DNAJ*, and *UBC2* presented the highest recommended frequency. In the leaves of *G. uralensis*, *CAC*, *DNAJ*, and *UBC2* were shared among three, three, and two experimental conditions tested, respectively, while *CAC*, *DNAJ*, and *UBC2* were selected as the top three most suitable reference gene by four, three, and three conditions in the root (Figure 4). In *G. inflata*, *COPS3* was the most recommended reference gene among the 11 genes appearing in the top three list and shared by four experimental conditions, including the leaves under normal condition, osmotic and salt stress, and MeJA treatment. Furthermore, *RAN*, *CAC*, and *CS* also showed higher recommended frequency and were shared among three experimental conditions in *G. inflata* (Figure 4). In addition to the genes mentioned above, much more reference genes in the top three list, such as *TUB*, *CYP*, and *TIF1* in *G. uralensis* and *R3HDM2* and *TIF1* in *G. inflata*, were only recommended by a single experimental condition (Figure 4).

**Figure 5:**
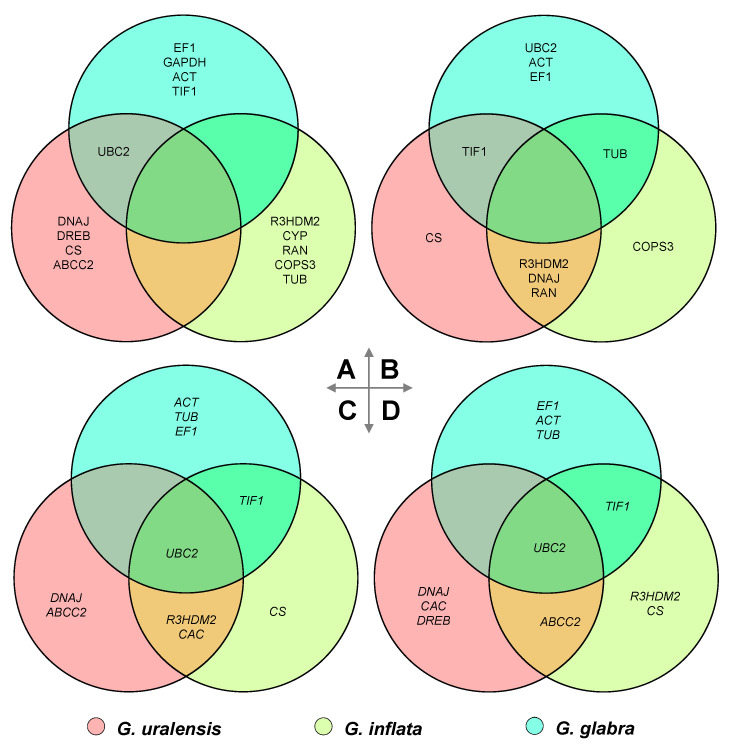
Comparison of the recommended reference genes among three *Glycyrrhiza* species: the Venn diagram compares the top five most suited reference genes recommended for the leaf (**A**,**B**) or root (**C**,**D**) under control or osmotic stress conditions, respectively, among the three congeneric species.

For comparison of the suitable reference genes among three congenic *Glycyrrhiza* species, the top five most suitable reference gene were considered. Results showed that no consistent reference gene was found among the leaves of these three species, *TIF1* was shared among *G. glabra* and *G. uralensis*, and *TUB* was shared among *G. glabra* and *G. inflata*, while *R3HDM2*, *DNAJ*, and *RAN* were selected as the top five most suitable reference gene by *G. uralensis* and *G. inflata* (Figure 5). In roots, *UBC2* was the only universal reference gene shared among *G. glabra*, *G. uralensis*, and *G. inflata* under drought stress, while *TIF1* or *UBC2* were shared among *G. glabra* and *G. inflata* or *G. glabra* and *G. uralensis*, respectively (Figure 5).

### 2.5. Validation of Recommended Reference Genes

*β-AS* (*β-amyrin synthase*) is a key gene in glycyrrhizin biosynthesis and mainly expressed in root or rhizome in licorice [27,28]. Previous studies showed that glycyrrhizin biosynthesis was induced by drought and MeJA treatment [22], and expression of *β-AS* was also upregulated by drought stress, which is in close correlation to glycyrrhizin accumulation [29]. We thus chose *β-AS* to validate the reliability of the selected reference genes under different conditions.

**Figure 6:**
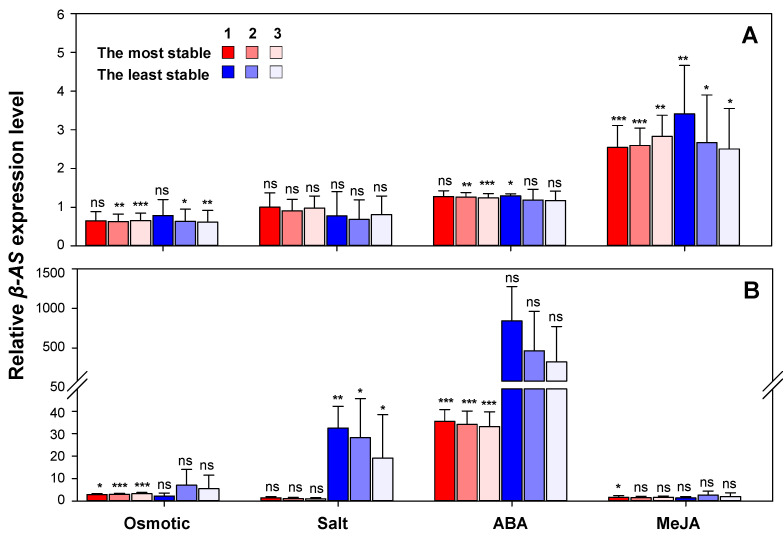
Validation of the recommended reference genes: expression profile of *β-AS* gene under different treatments relative to control in roots were investigated using the most/least suited, the top two most/least suited, and the top three most/least suited reference genes in *G. uralensis* (**A**) and *G. inflata* (**B**). Reference genes most suited or least suited for each species under different conditions are detailed in Table 1, Table S4, and Table S5. Bars represent the means ± standard deviation of biological replications. Results are presented as the fold change in relative expression compared to control using independent-samples T test method. * *p* < 0.05; ** *p* < 0.01; *** *p* < 0.001.

Results showed that, in both *G. uralensis* and *G. inflata*, *β-AS* expression profiles under all the experimental conditions were different when normalized to the most suitable and the least suitable reference genes, and the differences fall into three types: (1) *β-AS* expression was changed dramatically between control and stress conditions when using the most suitable candidate reference genes for normalization, but there was no obvious differences in the expression levels of *β-AS* when the least suitable reference gene were used (ABA treatment in *G. uralensis* and osmotic stress, and ABA and MeJA treatments in *G. inflata*). (2) *β-AS* expression had no obvious difference between control and stress conditions (*p* > 0.05) when using the most suitable candidate reference genes for normalization, while the difference became significant when the most unstable reference was used (*p* < 0.05) (*G. inflata* under salt stress). (3) The expression of *β-AS* presented significantly difference between control and stress conditions when normalized with the most and least reference genes, but the significance level of the differences was changed (*G. uralensis* under osmotic stress and MeJA treatment conditions) (Figure 6). All the results above demonstrated that, when analyzing gene expression before and after treatments and unsuitable reference genes used for normalization, significant expression alteration may be made insignificant or may disappear while unchanged expression may show a significant difference due to the altered expression of reference genes.

Moreover, different results were obtained when a different number of reference genes was used for normalization and it applied to both species. *β-AS* expression under same experimental conditions were different when normalized to the most, the two most, and the three most suitable candidate reference genes. Under ABA treatment in *G. uralensis*, when the most suitable reference gene (*CAC)* was used for normalization, the expression of *β-AS* under stress conditions showed no obvious differences (*p* < 0.05), but it changed dramatically in the expression levels of *β-AS* between control and stress conditions when using the two (*CAC* and *RAN*) or three most (*CAC*, *RAN*, and *UBC2*) suitable candidate reference genes for normalization (*p* < 0.01 or *p* < 0.001, respectively) (Figure 6). The same issues happened in *G. inflate* after MeJA treatment (Figure 6). Therefore, more than one reference gene may be required for accurate evaluation of gene expression.

### 2.6. Summary of Selected Reference Genes within the Leguminosae Plants

Reference gene selection has been reported in 18 Leguminosae species (*Ammopiptanthus mongolicus*, *Arachis hypogaea* L., *Cajanus cajan* (Linn.) Millsp., *Caragana intermedia*, *Caragana korshinskii* Kom, *Cassia obtusifolia* L., *Cicer arietinum* L., *Cyamopsis tetragonoloba* L.Taub, *Glycine max* (L.) Merr., *Glycyrrhiza glabra* L., *Oxytropis ochrocephala* Bunge, *Robinia pseudoacacia* L., *Trifolium repens* L., *Vigna angularis* (Willd.) Ohwi, *Vigna mungo* L., *Vigna unguiculata* L., *Medicago sativa* L., and *Pisum sativum* L.) under different experimental conditions, including abiotic stresses and hormonal treatments, by searching “NCBI” or “Web of Science” with the key words “Leguminosae” and “reference gene”. In this study, we added G. uralensis and G. inflata to this list.

**Figure 7:**
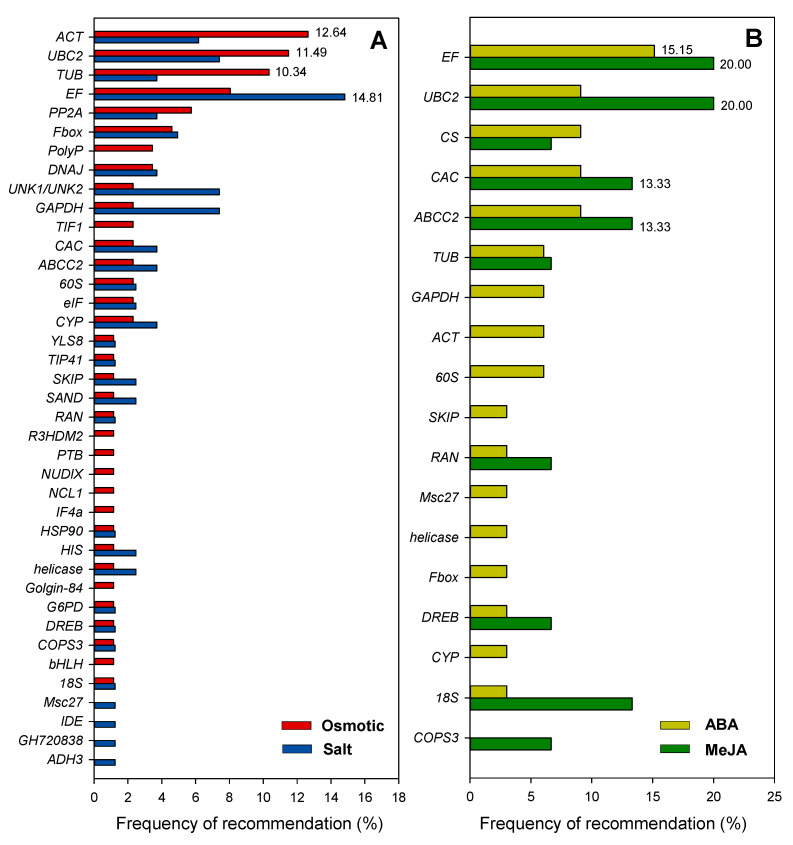
Frequency of reference genes recommended for qRT-PCR analysis under different experimental conditions in Leguminosae plants: here, we surveyed the frequency of each reference gene recommended for abiotic (osmotic/drought and salt) stresses (**A**) and hormonal (Abscisic acid (ABA) and methyl jasmonic acid (MeJA)) treatments (**B**) among Leguminosae species. The top three reference genes recommended for each species under different conditions are detailed in Table S5.

Eighteen species had been studied under osmotic or drought stress, among the 43 reference genes recommended: *actin* (*ACT*, *ACT11*, *ACT2*, and *ACT7*), *ubiquitin* (*UBC2*, *UBI1*, *UBQ*, *UBQ1*, and *Ubq28*), and *tubulin* (*TUA5*, *TUB*, *TUB4*, and *TUB6*) were the top three choices, and the frequency of recommendation was 12.64%, 11.49%, and 10.34%, respectively. In this study, our results further confirm this observation. When research involves the drought stress, *UBC2* can be used as the internal control for gene quantification studies among the three licorice species, *G. uralensis*, *G. inflata*, and *G. glabra* (Figure 7).

Moreover, *Eukaryotic elongation factor* was the optimal reference gene under salt stress, ABA, and MeJA treatments, with a recommendation frequency of 14.81% (*EF1A*, *EF1B*, *ELF-1α*, and *ELF1B*), 15.15% (*EF-1α*, *EF1α2*, and *ELF1b*), and 20% (*EF1A1a1*, *EF1A2a*, and *EF1A2b*), respectively (Figure 7). The expression of *EF1* in *G. uralensis* and *G. inflate*, however, was not stable throughout all the experimental conditions in this study (Figure 4).

## 3. Discussion

### 3.1. Stability of Candidate Reference Genes

The 14 candidate reference genes evaluated in the present study include 10 commonly used housekeeping gene in plants and 4 new generation candidate reference genes (*ABCC2*, *COPS3*, *CS,* and *R3HDM2*) selected form the RNA-Seq data of *Glycyrrhiza* species. In total, *CAC*, *UBC2,* and *ABCC2* were the three most stably expressed genes under different experimental conditions tested in the two *Glycyrrhiza* species. In the previous study, *CAC* was identified as the most stable reference gene in chickpea under drought stress condition [30]. In contrast, the expression of the *CAC* gene was the least stable under ABA treatment, osmotic stress, and cold stress [31]. In the present study, the *CAC* gene was most stable gene of *G. uralensis* under ABA and MeJA treatment, whereas *CAC* was the most stable gene of *G. inflata* under salt stress and ABA treatment (Figure 4 and Table 1). *UBC2* has been selected as the most stable reference gene in many species under various conditions, such as *G. glabra* under drought stress [26], and *A. mongolicus* and *V. unguiculata* under salt stress [32,33], while it was reported moderately stable in *A. mongolicus* under drought stress [32] and in *M. sativa* under salt stress and ABA treatment [34]. In this study, *UBC2* was identified as the most stable gene in *G. inflata* under ABA and MeJA treatment and as one of the top three most stable in *G. uralensis* under salt stress, ABA, and MeJA treatment (Figure 4 and Table 1), which is in agreement with the study in *A. mongolicus* and *V. unguiculata* [32,33]. *ABCC2* was selected as candidate based on the RNA-Seq data of *Glycyrrhiza* species. Results showed that they were the top three stable reference genes under many experimental conditions in *G. uralensis* and *G. inflata* (Figure 4 and Table 1), and it was the first time *ABCC2* was reported as the stable reference gene in Leguminosae species. Therefore, in addition to the housekeeping genes, stably expressed genes in RNA-seq experiments can be good candidates to search for reference genes.

*ACT*, which is the essential components of the eukaryotic cytoskeleton, has been reported to be the most stable gene in many plant species including *G. glabra* [26], *V. mungo* [35], and *R. pseudoacacia* [31]. However, its expression was variable under the tested experimental conditions of this study. Therefore, the housekeeping genes are not always stable in all the species, and under all the experimental conditions, it is necessary to confirm the expression of every reference gene used in each study.

### 3.2. Comparison of the Suitable Reference Genes under Different Experimental Conditions

In a parallel study, we have examined the optimal reference genes for different tissues in *G. uralensis* and *G. inflata* (Li et al., submitted). Results showed that the top three most suitable reference gene in *G. uralensis* and *G. inflata* were *R3HDM2*, *CAC*, and *TUB* and *COPS3*, *R3HDM2*, and *DREB*, respectively. In the present study, we evaluated the optimal internal reference genes of licorice under different treatment conditions, and the results showed that the optimal internal reference genes were different in root and shoot for the same conditions (Figure 4 and Table 1). More importantly, the optimal reference genes selected were different from those we screened for different tissues previously (Li et al., submitted). Therefore, we should choose different reference genes in different research. In addition, based on the stability rankings integrated by RefFinder in this study, the order from the most to the least stable candidate reference genes under different experimental conditions was not consistent (Figure 4 and Table 1), and no single gene was recommended by all the experimental conditions. The same situation also appeared in *G. inflata* (Figure 4 and Table 1) and a previous study (Li et al, submitted). The most stable reference genes were found to be *ACT* 7 and *TUB* under drought stress in *Cyamopsis tetragonoloba*, *GAPDH* and *EF-1a* under salt stress, *GAPDH* and *ACT 7* under heat stress, and *TUA* and *UBC2* under cold stress [36]. These results demonstrate that it is necessary to identify a suitable reference gene for each experimental condition.

This point is strongly supported when we analyzed the expression pattern of *β-AS*, a gene involved in the biosynthesis of glycyrrhizin, the major bioactive component in licorice root. When normalized to the most, the two most, and the three most suitable candidate reference genes, expression patterns were altered both in *G. uralensis* and *G. inflata* (Figure 6). Therefore, two or more reference genes may be necessary to avoid biased normalization under certain conditions. Besides, *β-AS* expression profiles under all the experimental conditions were different when normalized to (1) the most and least suited, (2) the two most and least suited, (3) the three most and least suited genes, and (4) all the candidate reference genes. Therefore, reliable reference genes are essential for the accurate normalization of gene expression levels.

### 3.3. Suitable Reference Genes for Glycyrrhiza Species under the Osmotic Stress

Our results showed the suitable reference genes were not universal among the three *Glycyrrhiza* species; *UBC2* was the only optimal reference gene shared among *G. glabra*, *G. uralensis,* and *G. inflata* under the osmotic stress (Figure 5, Table 1). For the leaves, *TIF1*, *TUB*, *R3HDM2*, *DNAJ*, and *RAN* were shared among two of the three *Glycyrrhiza* species, while no consistent reference gene was recommended among these three species simultaneously (Figure 5). Most of the recommended reference genes, such as *CS*, *COPS3*, *EF1*, and *ACT*, were selected by only one species (Figure 5 and Table 1). It is apparent that reference genes that worked perfectly in *G. glabra* may not work in *G. uralensis* or *G. inflata* and vice versa. Therefore, as mentioned above, housekeeping genes cannot be used as reference genes without validation, and several studies have reported variable expression of housekeeping genes under different conditions or species [37,38]. Use of improper reference genes can cause significant biases and misinterpretations of the expression data [39].

### 3.4. Summary of Recommended Reference Genes within the Leguminosae Plants

In this study, we also summarized the validated reference genes in Leguminosae species (including *G. uralensis* and *G. inflata* tested in this study) under abiotic stresses or hormonal treatments (Figure 7). From the results of our survey, we found that *ACT*, *TUB*, *UBQ*, and *EF* were the most optimal reference gene under salt stress, ABA treatment, and MeJA treatments. Among them, *ACT* was recommended as a stable reference gene under osmotic stress in *C. obtusifolia* [40], *C. tetragonoloba* [36], and *R. pseudoacacia* [31]. *EF* has been identified as the most suitable reference gene in *C. obtusifolia* [40], *G. max* [41], and *M. sativa* [34]. *TUB* was the optimal reference gene in *C. arietinum* [30], *G. max* [41], and *V. mungo* [35]. *UBQ* was validated as the most stable reference gene in *G. glabra* [26], and *V. unguiculata* [33]. Therefore, the optimal reference genes for different species in the same family are variable, even for the three proximal species in the same genus (*G. glabra*, *G. uralensis,* and *G. inflata*). We found *ACT* and *TIF1* were the most unstable reference genes in *G. uralensis* and *G. inflata*, and it has been proven that it will cause false results using the unstable reference gene for expression normalization (Figure 7). However, *ACT* is a commonly used housekeeping gene and has been identified as the most suitable reference genes in several studies [36,42,43]. Thus, it is always necessary to validate reference genes for reliable gene expression analysis and even housekeeping genes cannot be used as reference genes without validation [37,38]. This summary and analysis of the reported legume reference genes will serve as a guide for the subsequent selection of reference genes in Leguminosae plants.

## 4. Materials and Methods

### 4.1. Plant Materials and Treatments

Seeds (*G. uralensis* and *G. inflata*) used in this study were provided by Gansu Jin You Kang Pharmaceutical Technology Co., Ltd., Lanzhou, China. *Glycyrrhiza* seeds were soaked in H_2_SO_4_ (18 mol/L) for 30 min and then washed several times with water to remove the hard shell [29,44]. The washed seeds were treated with 1% NaClO for 10 min and washed five times with sterilized distilled water before planting. 

**Figure 8:**
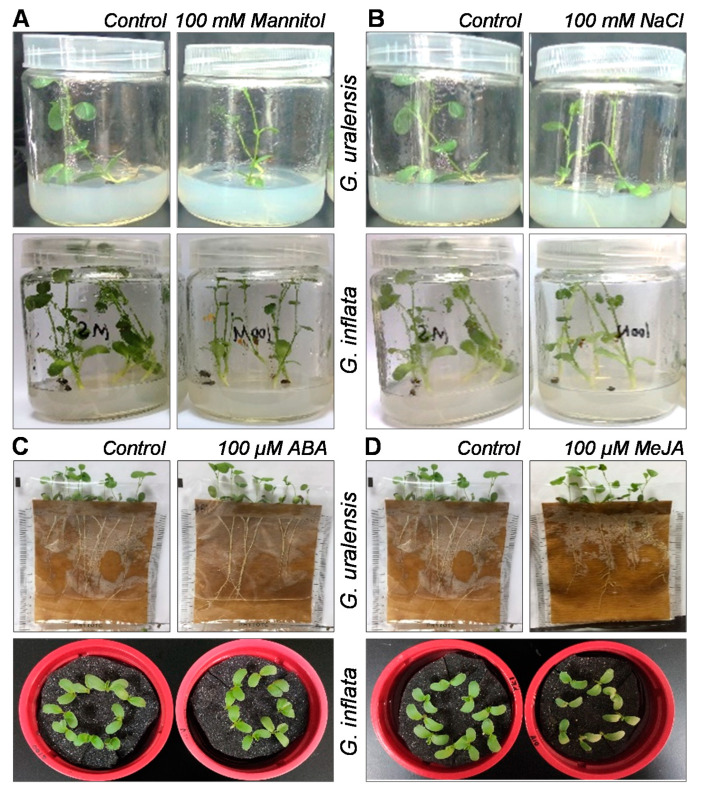
The morphology of *G. uralensis* and *G. inflata* under different experimental conditions: the morphology of *G. uralensis* and *G. inflata* under osmotic stresses (**A**), salt stresses (**B**), Abscisic acid (ABA) treatment (**C**), and methyl jasmonic acid (MeJA) treatment (**D**). Photos were taken 4 weeks after osmotic and salt treatments and 6 h after ABA or MeJA treatments.

Seeds used for osmotic or salt stress treatment were sown on Murashige and Skoog (MS) medium plates with or without 100 mM mannitol (osmotic stress) or 100 mM NaCl (salt stress) and grown for four weeks. Leaves and roots of every 20 seedlings were harvested with liquid nitrogen as one sample and stored at −80 °C (Figure 8). The seeds used for hormonal treatment were put on wet filter paper until the hypocotyls were 1–2 cm long and then transferred into Hoagland’s nutrient solution. The nutrient solution was changed every 2–3 days. Fourteen days later, ABA (ABA treatment) or methyl JA (MeJA treatment) were added to the solution to make the final concentration 100 μM, and 0.1% ethanol in solution was used as a control. After 6 h, leaves or roots of every 10 seedlings were harvested with liquid nitrogen as one sample and stored at −80 °C (Figure 8). All plants were grown in a growth room at 24 °C with a photoperiod cycle of 14 h light 10 h dark. All experiments were conducted with three biological replicates.

### 4.2. Stability Analysis of Candidate Reference Genes

Total RNA was isolated using the HiPure Total RNA Mini Kit (Magen, Shanghai, China, Cat. R4151-03) according to the kit instructions. The RNA quality and concentration were measured with agarose gel electrophoresis and a spectrophotometer (NanoDrop 2000, Thermo Scientific, New York, USA). Removal of genomic DNA contamination and first strand cDNA synthesis were performed using the PrimeScript^TM^ RT reagent Kit with gDNA Eraser (TaKaRa, Kyoto, Japan, Cat. RR047A) following the manufacturer’s instructions. Quantitative PCR were carried out in 384-well blocks using TB Green™ Premix Ex Taq™ II (Tli RNaseH Plus) (TaKaRa, Japan, Cat. RR820D) on LightCycler 480 (Roche, Basel, Switzerland) according to manufacturers’ instructions. 

A total of 14 candidates were selected, and the stability was evaluated. Ten commonly used candidate housekeeping genes (*ACT*, *CAC*, *CYP*, *DNAJ*, *DREB*, *EF1*, *RAN*, *TIF1*, *TUB*, and *UBC2*) were selected from published literature [26,45]. Protein sequences of these candidates were downloaded from the NCBI database; nucleotide sequences of these genes were obtained from *G. uralensis* genome (http://ngs-data-archive.psc.riken.jp/Gur-genome/index.pl#) and *G. inflata* RNA-Seq database (SRA accession: PRJNA574093) using TBLASTN program in BioEdit software. Four new generation candidate genes (*ABCC2*, *COPS3*, *CS,* and *R3HDM2*) were selected from the RNA-seq data and expressed consistently in different RNA-Seq samples. qRT-PCR primers were designed using PrimerQuest Tool, INTEGRATED DNA TECHNOLOGIES (IDT) (https://sg.idtdna.com/Primerquest/Home/Index). All primers were synthesized by TSINGKE (Guangzhou, China). The primer information used in this study were listed in Table 2.

Expression stability of the 14 candidate reference genes were evaluated by four Microsoft Excel-based computational programs, geNorm [46], NormFinder [47], BestKeeper [48], and Delta CT [49]. geNorm ranks genes by stability values (M value); the most stable reference gene is the one having the lowest M value. Pairwise variation analysis (Vn/*n*+1), for investigating the optimal number of reference genes for accurate normalization, was also performed by geNorm software. The threshold was commonly set at 0.15, and additional reference genes are not required when Vn/*n*+1 is below 0.15. NormFinder identifies suitable reference genes on the basis of stability value (SV); the candidate having the lowest SV is the optimal reference gene. BestKeeper and Delta CT rank the stabilities of candidates on the basis of SD and CV values; the most stable reference gene is the one having the lowest SD value.

The comprehensive ranking of expression stability of candidate reference genes were conducted using RefFinder, which is a system to integrate results obtained by the geNorm, Normfinder, BestKeeper, and Delta Ct method [50]. Based on the rankings from the Microsoft Excel-based computational programs, RefFinder assigns an appropriate weight to an individual gene and calculates the geometric mean of their weights for the overall final ranking. 

### 4.3. Comparison of the Suitable Reference Genes under Different Experimental Conditions

The top three most suitable reference genes selected by RefFinder under the conditions of osmotic stress, salt stress, ABA treatment, and MeJA treatment were compared; the suitable reference genes under osmotic stress condition among the three congenic *Glycyrrhiza* species were also analyzed. The results were visualized by Venn Diagrams, and it was plotted using the OmicShare tools, a free online platform for data analysis (www.omicshare.com/tools).

### 4.4. Survey of the Reference Genes Used within the Leguminosae Plant

Survey of the reference genes used within Leguminosae plants was conducted by searching keywords of “Leguminosae” and “reference” on Web of Science and PubMed. A total of 18 different Leguminosae species under various experimental conditions, including abiotic stresses and hormonal treatments, were included in the analysis. The top three most suitable reference genes for any given Leguminosae species under specific experimental condition (osmotic/salt stress or ABA/MeJA treatment) were used to calculate the frequency of recommendation.

## 5. Conclusions

In this study, the leaves and roots of two *Glycyrrhiza* species under four experimental conditions (osmotic stress, salt stress, ABA treatment, and MeJA treatment) were examined for gene expression in order to identify reliable reference genes. Different reference genes were identified in different tissues under different experimental conditions as well as in different species. In addition, we also documented the reference genes that have been used in qRT-PCR analysis among 18 different Leguminosae plants under the same abiotic conditions with current study, i.e., osmotic/salt stress and ABA/MeJA treatment. Among the 132 genes tested, even the routinely used reference genes showed variable expressions under different experimental conditions. Therefore, accurate measurements of gene expression for functional characterization require suitable reference genes to avoid misinterpretation of qRT-PCR results, and a thorough evaluation of reference genes is strongly recommended.

## Figures and Tables

**Table 1:** The top three recommended reference genes under different experimental conditions in *G. uralensis* and *G. inflata*.

Conditions	*G. uralensis*		*G. inflata*	
	Leaf	Root	Leaf	Root
Control	*CS, DNAJ, DREB*	*DNAJ, UBC2, CAC*	*COPS3, TUB, RAN*	*CS, CAC, R3HDM2*
Osmotic stress	*TIF1, DNAJ, RAN*	*DNAJ, CAC, DREB*	*TUB, COPS3, DNAJ*	*TIF1, R3HDM2, ABCC2*
Salt stress	*DNAJ, CYP, CAC*	*DREB, DNAJ, UBC2*	*CAC, RAN, COPS3*	*ABCC2, CAC, DNAJ*
ABA treatment	*CS, CAC, UBC2*	*CAC, RAN, UBC2*	*UBC2, CS, DREB*	*CAC, TUB, CS*
MeJA treatment	*ABCC2, UBC2, CAC*	*CAC, TUB, ABCC2*	*RAN, COPS3, UBC2*	*UBC2, DREB, CS*

**Table 2:** Primers used in this study.

Gene	Description	Accession Number	Primer Sequence (5’–3’) Forward/Reverse	Amplicon Length (bp)	Tm (°C)	E (%) *
*ACT*	*Actin1*	MW119712	CCCACTCAACCCAAAGGC/TAACCCTCATAGATTGGCACAG	183	62.8	92.72
*CAC*	*Clathrin complex AP1*	MW116276	GAGTTTCAGCTTCCTCCTTGCA/TGATGGGGCTTTATCCTTTGG	126	63.4	116.84
*CYP*	*Cyclophilin*	MW119709	AAGACGGAGTGGCTGGACG/TCTTGCCGGAGCTGGACC	103	67	92.9
*DNAJ*	*Heat-shock protein 40*	MW116277	TGGTTGTCAAGGAACTGGTATG/CACTGTGGGCAGCGGTCT	135	63.4	91.94
*DREB*	*Dehydration responsive element binding*	MW119710	GGTTGCTGAAATTCGGGAGC/CATTGGGGAAGTTGAGGCG	139	64	97.83
*EF1*	*Translation elongation factor1*	MW116273	GACTGGTACAAGGGACCAAC/AGACATCCTGCAATGGAAGC	101	63.1	90.42
*RAN*	*Ras related protein*	MW116274	ACAGAGCAGACGATGACTACGA/CTGAGCCTTGATGACTTTGGA	185	63.2	91.22
*TIF1*	*Translation initiation factor*	MW122063	ACAACCGTTCAGGGATTGA/GGGTCCTGAACAACTGTACC	98	62.2	77.95
*TUB*	*β-Tubulin*	MW119713	CCTTGAGCCAGGCACCAT/GTCCTTTCGCCCAGTTGTT	113	63.6	86.97
*UBC2*	*Ubiquitin-conjugating enzyme E2*	MW116271	CTTCAACAAGACCCACCTGC/ACGTGCCTCCATCCCATG	112	64.1	93.51
*ABCC2*	*ATP binding-box transporter 2*	MW116275	TGAGTCTTTCCAGGGCTTTATT/ATGGTGTTAAGGCGATGAGC	160	62.7	90.63
*COPS3*	*COP9 signal complex subunit 3*	MW119711	GGAAGCGCCAATACGAGG/ACAACAAGCACAGCAGAAGAAA	113	63.4	92.32
*CS*	*Citrate synthase*	MW116272	GCTCAGCCGTTGACCCAG/CACCACCAGGAAAAGCACC	93	64.2	107.58
*R3HDM2*	*R3H domain protein 2*	MW119714	GCTTTGGGTTCAATGGAGG/TCAGCAGAGTGCTGGGGTC	115	61.9	98.12

“*”: Amplification efficiency.

## Data Availability

Data will be available upon request. RNA-seq datasets used in this study can be found in online repositories. The names of the repository/repositories and accession number(s) can be found below: https://www.ncbi.nlm.nih.gov/, PRJNA574093.

## Supplementary Materials

The following are available at https://www.mdpi.com/2223-7747/9/11/1441/s1: Table S1: All the Cq Values under different experimental conditions in *G. uralensis*, Table S2: All the Cq Values under different experimental conditions in *G. inflata*, Table S3: Cq values of candidate reference genes under different conditions in *G. uralensis* and *G. inflata*, Table S4: Stability of candidate reference genes under different conditions in *G. uralensis*, Table S5: Stability of candidate reference genes under different conditions in *G. inflata*, Table S6: Recommended reference genes for qRT-PCR analysis under different conditions in Leguminosae plants.
