# Supplementary material for: Selection of Reference Genes for qRT-PCR Analysis in Medicinal Plant Glycyrrhiza under Abiotic Stresses and Hormonal Treatments
Source: Plants (Basel). 2020 Oct 26;9(11):1441. doi: 10.3390/plants9111441 (PMC7692165; doi:10.3390/plants9111441)
Supplement: Supplementary file 1 [file plants-09-01441-s001.pdf]

Selection of reference genes for qRT-PCR analysis in *Glycyrrhiza* species under abiotic stresses and hormonal treatments

Yuping Li[1, 3], Xiaoju Liang[1, 4], Xuguo Zhou[5], Zhigeng Wu[1, 4], Ling Yuan[1, 3, 6], Ying Wang[1, 2, 3, *], Yongqing Li[1, 3,*]

[1] Key Laboratory of South China Agricultural Plant Molecular Analysis and Genetic Improvement & Guangdong Provincial Key Laboratory of Applied Botany, South China Botanical Garden, Chinese Academy of Sciences, Guangzhou 510650, China.
[2] Gannan Normal University, Ganzhou, Jiangxi, 341000, P.R. China.
[3] Center of Economic Botany, Core Botanical Gardens, Chinese Academy of Sciences, Guangzhou, 510650, China.
[4] University of Chinese Academy of Sciences, Beijing 100049, China.
[5] Department of Entomology, University of Kentucky, Lexington, KY 40546, USA.
[6] Department of Plant and Soil Sciences, University of Kentucky, Lexington, KY 40546, USA.

---

[*] For correspondence
yingwang@scib.ac.cn (Y. Wang)
liyongqing@scbg.ac.cn (Y. Li)

Table S1. All the Cq Values under different experimental conditions in *G.uralensis*.

Conditions	*ACT*	*CAC*	*CYP*	*DNAJ*	*DREB*	*EF1*	*RAN*	*TIF1*	*TUB*	*UBC2*	*ABCC2*	*COPS3*	*CS*	*R3HDM2*
**Abiotic stresses**														
Control_Leaf_1	19.89	23.70	22.51	21.19	24.82	23.81	24.48	17.30	23.61	20.71	21.12	25.63	23.11	23.61
Control_Leaf_2	25.11	27.63	25.58	24.77	27.67	29.57	27.79	21.81	27.58	24.25	25.47	25.95	26.19	27.56
Control_Leaf_3	26.57	25.74	24.92	23.03	26.40	28.80	25.90	21.10	28.06	22.49	24.45	26.06	25.02	27.79
Control_Root_1	24.64	27.85	24.60	24.58	28.27	27.71	27.40	21.65	26.26	24.02	24.24	24.25	25.74	26.88
Control_Root_2	22.90	26.23	22.93	23.10	26.71	26.64	25.66	20.01	24.64	22.46	22.70	24.97	23.98	25.22
Control_Root_3	25.65	27.83	25.55	24.71	28.42	28.62	27.90	22.60	26.76	24.05	23.96	25.53	25.61	26.62
Osmotic stress_Leaf_1	20.12	23.97	21.63	21.29	24.78	23.76	23.87	18.24	22.74	20.98	21.29	23.39	22.97	23.21
Osmotic stress_Leaf_2	27.11	27.71	25.61	25.07	27.78	30.50	27.69	22.71	28.40	24.85	26.52	26.83	27.01	28.52
Osmotic stress_Leaf_3	29.13	29.41	28.02	29.29	27.95	35.00	32.36	26.40	34.74	26.90	31.32	27.50	31.67	32.06
Osmotic stress_Root_1	22.97	26.26	23.65	23.13	27.08	26.05	25.92	19.91	24.68	22.66	22.74	25.69	24.33	25.19
Osmotic stress_Root_2	24.23	27.63	24.53	24.63	28.36	28.21	27.25	22.28	25.85	24.30	24.12	25.15	25.59	26.40
Osmotic stress_Root_3	22.10	25.04	22.17	21.87	25.62	25.12	24.59	19.84	23.58	21.55	21.80	25.03	22.78	24.46
Salt stress_Leaf_1	19.63	23.84	21.80	20.97	24.71	23.56	23.98	17.92	22.76	20.90	21.09	23.32	22.75	22.99
Salt stress_Leaf_2	22.88	26.12	24.58	23.49	26.13	26.87	26.06	20.01	24.75	22.64	23.45	26.40	24.65	25.80
Salt stress_Leaf_3	25.43	25.79	24.16	22.99	26.52	27.73	25.65	21.34	25.73	22.57	23.38	29.99	24.30	26.46
Salt stress_Root_1	24.28	27.44	24.24	24.00	28.12	27.15	27.37	21.69	25.51	23.83	23.00	25.10	24.96	25.64
Salt stress_Root_2	22.36	27.88	25.04	24.84	28.62	29.26	27.65	22.22	26.55	24.55	24.67	26.30	25.81	27.17
Salt stress_Root_3	25.72	25.79	25.97	25.05	28.96	29.14	28.44	23.20	26.76	24.91	24.37	24.65	25.78	26.84
**Hormonal treatments**														
Control_Leaf_1	21.97	25.23	21.93	22.29	25.67	26.47	24.22	19.75	23.24	21.46	22.03	24.81	23.53	24.91
Control_Leaf_2	22.09	24.74	21.96	21.42	24.26	26.33	23.02	18.72	23.91	20.35	21.68	23.83	22.73	24.87

Control_Leaf_3	22.58	25.80	22.62	23.09	26.12	26.06	24.92	20.08	23.30	21.73	22.21	23.58	23.67	24.93
Control_Root_1	21.04	26.12	24.15	22.77	26.18	27.14	25.27	20.56	24.20	22.15	22.81	25.86	24.08	25.58
Control_Root_2	23.26	25.86	22.79	22.72	26.28	26.47	25.21	20.69	23.71	22.11	22.07	24.48	23.16	25.09
Control_Root_3	25.00	27.86	24.85	24.64	28.23	27.68	27.29	22.74	25.08	24.03	23.89	24.86	24.95	26.49
ABA treatment_Leaf_1	22.49	25.72	22.44	22.60	25.94	25.98	24.34	19.68	23.34	21.56	22.56	24.47	23.68	25.28
ABA treatment_Leaf_2	22.08	25.52	22.60	22.66	25.91	26.65	24.49	19.58	23.52	21.58	22.46	24.81	23.70	25.33
ABA treatment_Leaf_3	22.93	26.04	23.02	23.17	26.22	27.23	24.98	19.94	23.82	21.96	22.95	24.62	24.12	25.70
ABA treatment_Root_1	22.43	25.97	22.94	22.16	25.87	27.15	25.28	20.03	24.63	21.90	23.11	24.75	23.83	25.51
ABA treatment_Root_2	23.31	26.90	23.78	23.31	27.12	28.36	26.03	20.92	25.10	22.79	24.09	24.66	24.73	25.91
ABA treatment_Root_3	25.18	28.51	26.12	24.91	28.82	29.29	27.92	23.70	26.04	24.90	24.54	25.75	25.56	26.75
MeJA treatment_Leaf_1	23.74	26.46	23.71	22.89	26.05	27.03	25.63	19.50	25.25	21.90	22.99	24.50	24.02	26.00
MeJA treatment_Leaf_2	22.12	25.55	22.57	22.00	25.37	26.15	24.57	19.37	23.69	21.10	21.82	26.94	22.52	24.71
MeJA treatment_Leaf_3	23.91	26.87	23.63	23.47	26.87	27.58	26.03	20.94	24.74	22.73	22.93	23.74	23.85	25.64
MeJA treatment_Root_1	22.44	25.82	22.74	23.02	25.53	26.91	24.96	19.69	23.71	21.81	22.72	24.82	24.07	25.99
MeJA treatment_Root_2	22.31	25.64	22.70	22.59	25.44	26.33	24.41	19.89	23.17	21.61	22.04	24.47	23.49	24.84
MeJA treatment_Root_3	23.39	26.08	23.32	23.47	26.32	26.98	25.27	20.72	24.02	22.46	22.91	24.54	24.27	25.60

Table S2. All the Cq Values under different experimental conditions in *G. inflata*.

Conditions	*ACT*	*CAC*	*CYP*	*DNAJ*	*DREB*	*EF1*	*RAN*	*TIF1*	*TUB*	*UBC2*	*ABCC2*	*COPS3*	*CS*	*R3HDM2*
**Abiotic stresses**														
Control_Leaf_1	22.36	25.47	23.35	22.58	25.98	25.33	24.97	19.48	24.02	20.95	22.52	23.41	23.80	23.58
Control_Leaf_2	23.65	25.67	22.91	22.85	26.23	25.02	24.93	20.55	24.26	21.46	22.47	23.52	24.21	24.23
Control_Leaf_3	23.53	25.21	23.34	22.72	24.68	24.82	24.65	19.09	23.88	20.07	20.89	23.23	22.64	23.76
Control_Root_1	19.86	26.98	25.22	22.47	26.42	25.24	25.95	20.30	26.17	22.21	21.88	23.99	23.72	24.09
Control_Root_2	19.21	26.31	23.76	22.79	25.18	23.93	25.98	19.56	24.69	21.58	21.18	23.27	22.85	23.80
Control_Root_3	20.00	25.81	23.81	22.32	26.37	23.91	26.13	19.87	23.75	22.18	20.97	23.55	22.64	24.00
Osmotic stress_Leaf_1	17.76	24.90	22.12	22.06	25.43	24.15	23.36	18.48	22.97	21.22	21.90	22.87	23.28	23.02
Osmotic stress_Leaf_2	18.19	24.91	22.64	22.43	27.09	26.08	24.16	20.02	23.41	22.51	23.01	23.90	24.20	24.05
Osmotic stress_Leaf_3	24.24	26.27	23.51	23.20	26.23	24.68	25.11	19.18	24.18	20.58	22.47	23.03	23.58	23.38
Osmotic stress_Root_1	21.03	25.01	22.91	20.80	24.81	23.83	24.37	18.84	23.94	20.93	20.53	22.83	22.49	23.02
Osmotic stress_Root_2	20.67	25.51	22.69	21.26	24.72	22.72	24.02	18.51	22.31	21.00	20.59	21.79	22.51	22.63
Osmotic stress_Root_3	18.81	24.86	22.68	21.46	24.57	23.12	25.07	18.56	22.96	20.80	20.27	22.53	22.30	22.72
Salt stress_Leaf_1	21.61	25.06	22.56	22.54	27.21	24.41	24.02	24.89	23.21	22.77	23.54	23.18	24.97	23.47
Salt stress_Leaf_2	19.55	27.02	24.87	24.28	27.64	26.31	25.80	26.50	25.06	24.01	24.91	24.72	26.51	24.87
Salt stress_Leaf_3	17.65	26.77	24.56	24.22	27.18	23.90	25.83	24.63	25.10	22.66	23.32	22.82	24.31	23.14
Salt stress_Root_1	25.16	26.31	25.28	22.65	27.14	23.82	25.68	25.14	24.67	24.29	22.26	22.94	24.24	23.06
Salt stress_Root_2	18.48	25.54	23.94	21.71	25.67	23.95	24.94	24.90	23.92	21.26	21.55	22.75	23.55	23.01
Salt stress_Root_3	24.17	25.84	24.00	22.38	21.76	23.48	25.04	24.46	23.14	22.56	21.53	22.39	23.09	22.67
**Hormonal treatments**														
Control_Leaf_1	17.55	26.10	22.26	23.37	24.76	23.04	24.31	18.99	22.80	20.72	21.94	22.38	22.83	23.74
Control_Leaf_2	17.95	26.16	22.62	23.86	25.39	23.46	24.65	18.71	23.18	21.09	22.72	22.55	23.52	23.02
Control_Leaf_3	16.72	24.85	21.47	22.20	23.95	22.86	23.20	17.60	22.27	19.74	21.08	21.87	22.25	23.29
Control_Root_1	20.10	25.24	22.31	21.92	24.84	21.70	24.49	19.62	22.29	21.00	20.17	21.79	21.90	22.93

Control_Root_2	19.51	26.72	22.18	23.92	26.29	23.17	26.34	20.24	24.11	22.69	22.13	22.52	23.18	23.29
Control_Root_3	17.94	23.46	20.81	20.17	23.87	20.06	22.81	17.18	21.24	19.77	19.59	20.29	20.55	21.28
ABA treatment_Leaf_1	18.81	24.93	23.31	24.23	24.83	25.21	25.25	19.14	24.08	20.51	21.78	22.91	23.07	24.13
ABA treatment_Leaf_2	18.01	26.59	23.01	24.09	24.61	24.59	24.91	18.18	23.98	20.57	21.80	22.81	23.19	24.05
ABA treatment_Leaf_3	20.29	25.53	22.22	22.92	24.83	25.20	23.96	19.63	22.67	20.48	21.81	23.18	23.00	24.46
ABA treatment_Root_1	24.72	25.62	22.30	22.89	24.13	23.99	24.62	20.05	23.12	20.47	19.85	22.63	21.65	23.51
ABA treatment_Root_2	24.50	25.85	22.44	23.00	25.57	23.46	24.53	19.69	23.09	21.36	21.00	22.61	22.54	22.58
ABA treatment_Root_3	23.00	25.41	22.50	22.54	25.41	22.06	24.09	20.11	22.51	21.58	21.10	21.47	22.24	22.27
MeJA treatment_Leaf_1	23.40	25.64	21.71	23.06	25.28	23.96	23.90	18.79	22.58	20.17	20.84	22.21	21.82	23.63
MeJA treatment_Leaf_2	22.92	25.14	21.57	22.71	25.72	24.28	23.53	18.45	22.16	20.58	21.28	22.78	22.73	23.88
MeJA treatment_Leaf_3	22.85	26.56	23.06	24.02	25.33	24.06	24.75	19.11	23.80	21.01	22.10	23.33	23.28	24.67
MeJA treatment_Root_1	19.21	25.46	20.47	22.17	24.65	22.87	24.66	19.53	23.64	21.16	20.62	22.28	21.63	20.62
MeJA treatment_Root_2	19.78	24.81	20.95	22.53	24.77	22.96	25.07	19.23	22.11	21.12	20.84	22.02	22.51	22.88
MeJA treatment_Root_3	19.55	24.78	20.49	21.34	24.44	22.44	24.26	18.93	22.89	20.97	20.60	21.89	21.84	22.81

Table S3 Cq values of candidate reference genes under different conditions in *G. uralensis* and *G. inflata*.

Conditions	*ACT*	*CAC*	*CYP*	*DNAJ*	*DREB*	*EF1*	*RAN*	*TIF1*	*TUB*	*UBC2*	*ABCC2*	*COPS3*	*CS*	*R3HDM2*
***G. uralensis*_abiotic stresses**														
Control_Leaf	23.85±1.17	25.69±0.66	24.34±0.54	23.00±0.60	26.29±0.48	27.40±1.04	26.06±0.55	20.07±0.81	26.41±0.81	22.48±0.59	23.68±0.76	25.88±0.08	24.77±0.52	26.32±0.78
Control_Root	24.40±0.46	27.30±0.31	24.36±0.44	24.13±0.30	27.80±0.31	27.66±0.33	26.98±0.39	21.42±0.44	25.89±0.37	23.51±0.30	23.63±0.27	24.92±0.22	25.11±0.33	26.24±0.30
Osmotic_Leaf	25.46±1.58	27.03±0.93	25.08±1.08	25.22±1.33	26.83±0.59	29.75±1.89	27.97±1.42	22.45±1.36	28.63±2.00	24.24±1.00	26.37±1.67	25.91±0.73	27.22±1.45	27.93±1.48
Osmotic_Root	23.10±0.36	26.31±0.43	23.45±0.40	23.21±0.46	27.02±0.46	26.46±0.53	25.92±0.44	20.67±0.46	24.70±0.38	22.84±0.46	22.89±0.39	25.29±0.12	24.23±0.47	25.35±0.33
Salt_Leaf	22.65±0.97	25.25±0.41	23.51±0.50	22.49±0.44	25.79±0.32	26.05±0.73	25.23±0.37	19.75±0.57	24.41±0.50	22.04±0.33	22.64±0.45	26.57±1.11	23.90±0.34	25.09±0.61
Salt_Root	24.12±0.56	27.03±0.37	25.09±0.29	24.63±0.19	28.57±0.14	28.52±0.39	27.82±0.18	22.37±0.26	26.28±0.22	24.43±0.18	24.01±0.30	25.35±0.28	25.52±0.16	26.55±0.27
***G. uralensis*_hormonal treatments**														
Control_Leaf	22.21±0.11	25.26±0.18	22.17±0.13	22.27±0.28	25.35±0.32	26.29±0.07	24.05±0.32	19.52±0.24	23.48±0.12	21.18±0.25	21.97±0.09	24.07±0.22	23.31±0.17	24.91±0.01
Control_Root	23.10±0.66	26.61±0.36	23.93±0.35	23.38±0.37	26.90±0.38	27.10±0.20	25.92±0.40	21.33±0.41	24.33±0.23	22.76±0.37	22.92±0.31	25.07±0.24	24.07±0.30	25.72±0.24
ABA_Leaf	22.50±0.14	25.76±0.09	22.69±0.10	22.81±0.11	26.02±0.06	26.62±0.21	24.60±0.11	19.73±0.06	23.56±0.08	21.70±0.08	22.66±0.09	24.63±0.06	23.83±0.08	25.44±0.08
ABA_Root	23.64±0.47	27.13±0.43	24.28±0.55	23.46±0.46	27.27±0.49	28.27±0.36	26.41±0.45	21.55±0.64	25.26±0.24	23.20±0.51	23.92±0.24	25.05±0.20	24.71±0.29	26.05±0.21

MeJA_Leaf	23.26±0.33	26.29±0.22	23.31±0.21	22.79±0.25	26.10±0.25	26.92±0.24	25.41±0.25	19.94±0.29	24.56±0.27	21.91±0.27	22.58±0.22	25.06±0.56	23.47±0.27	25.45±0.22
MeJA_Root	22.72±0.20	25.85±0.07	22.92±0.12	23.03±0.15	25.76±0.16	26.74±0.12	24.88±0.15	20.10±0.18	23.63±0.14	21.96±0.15	22.56±0.15	24.61±0.06	23.95±0.13	25.48±0.19
***G. inflata*_abiotic stresses**														
Control_Leaf	23.18±0.24	25.45±0.08	23.20±0.08	22.72±0.05	25.63±0.28	25.05±0.09	24.85±0.06	19.71±0.25	24.05±0.06	20.83±0.23	21.96±0.31	23.38±0.05	23.55±0.27	23.85±0.11
Control_Root	19.69±0.14	26.37±0.20	24.27±0.28	22.53±0.08	25.99±0.23	24.36±0.25	26.02±0.03	19.91±0.12	24.87±0.41	21.99±0.12	21.34±0.16	23.61±0.12	23.07±0.19	23.96±0.05
Osmotic_Leaf	20.06±1.21	25.36±0.26	22.76±0.23	22.56±0.20	26.25±0.28	24.97±0.33	24.21±0.29	19.22±0.26	23.52±0.20	21.44±0.33	22.46±0.19	23.26±0.19	23.69±0.16	23.48±0.17
Osmotic_Root	20.17±0.4	25.13±0.11	22.76±0.04	21.17±0.11	24.70±0.04	23.22±0.19	24.49±0.18	18.64±0.06	23.07±0.27	20.91±0.03	20.46±0.06	22.39±0.18	22.43±0.04	22.79±0.07
Salt_Leaf	19.6±0.66	26.28±0.36	24.00±0.42	23.68±0.33	27.34±0.09	24.88±0.42	25.22±0.35	25.34±0.34	24.46±0.36	23.15±0.25	23.93±0.29	23.58±0.34	25.26±0.38	23.83±0.31
Salt_Root	22.60±1.20	25.90±0.13	24.41±0.25	22.25±0.16	24.86±0.93	23.75±0.08	25.22±0.13	24.83±0.12	23.91±0.26	22.70±0.51	21.78±0.14	22.69±0.09	23.63±0.19	22.92±0.07
***G. inflata*_hormonal treatments**														
Control_Leaf	17.40±0.21	25.70±0.25	22.12±0.19	23.14±0.28	24.70±0.24	23.12±0.10	24.05±0.25	18.43±0.24	22.75±0.15	20.51±0.23	21.91±0.27	22.27±0.12	22.87±0.21	23.35±0.12
Control_Root	19.18±0.37	25.14±0.55	21.77±0.28	22.00±0.63	25.00±0.41	21.64±0.52	24.54±0.59	19.01±0.54	22.55±0.48	21.15±0.49	20.63±0.44	21.53±0.38	21.88±0.44	22.50±0.36
ABA_Leaf	19.04±0.39	25.68±0.28	22.85±0.19	23.75±0.24	24.75±0.04	25.00±0.12	24.71±0.22	18.98±0.24	23.58±0.26	20.52±0.01	21.79±0.00	22.97±0.06	23.08±0.03	24.21±0.07
ABA_Root	24.08±0.31	25.63±0.07	22.41±0.03	22.81±0.08	25.04±0.26	23.17±0.33	24.41±0.09	19.95±0.08	22.91±0.11	21.14±0.20	20.65±0.23	22.24±0.22	22.14±0.15	22.79±0.22

MeJA_Leaf	23.06± 0.10	25.78± 0.24	22.11± 0.28	23.26± 0.22	25.44± 0.08	24.10± 0.05	24.06± 0.21	18.78± 0.11	22.85± 0.28	20.59± 0.14	21.41± 0.21	22.77± 0.19	22.61± 0.25	24.06± 0.18
MeJA_Root	19.51± 0.10	25.02± 0.13	20.64± 0.09	22.01± 0.20	24.62± 0.06	22.76± 0.09	24.66± 0.14	19.23± 0.10	22.88± 0.26	21.09± 0.03	20.69± 0.05	22.06± 0.07	21.99± 0.15	22.10± 0.43

Table S4 Stability of candidate reference genes under different conditions in *G. uralensis*.

Candidate genes	geNorm		NormFinder		BestKeeper		ΔCt method		RefFinder method	
	Stability	Ranking	Stability	Ranking	Stability	Ranking	Stability	Ranking	Stability	Ranking
**Control_Leaf**										
ACT	0.935	13	1.298	14	1.87	13	1.43	13	13.24	14
CAC	0.421	5	0.7	9	0.92	3	0.95	9	5.9	6
CYP	0.607	8	0.477	5	1.33	9	0.89	6	6.82	8
DNAJ	0.313	1	0.623	6	1	4	0.89	4	3.13	2
DREB	0.313	1	0.638	8	0.91	2	0.9	7	3.25	3
EF1	0.762	11	0.888	11	1.56	12	1.12	11	11.24	12
RAN	0.494	6	0.704	10	1.19	7	0.97	10	8.05	11
TIF1	0.652	9	0.625	7	1.2	8	0.95	8	7.97	10
TUB	0.855	12	1.142	12	1.91	14	1.32	12	12.47	13
UBC2	0.335	3	0.44	3	1.02	5	0.8	3	3.41	4
ABCC2	0.562	7	0.192	1	1.42	11	0.79	2	3.52	5
COPS3	1.007	14	1.292	13	0.9	1	1.44	14	7.1	9
CS	0.368	4	0.277	2	1.04	6	0.76	1	2.63	1
R3HDM2	0.695	10	0.465	4	1.37	10	0.89	5	6.69	7
**Control_Root**										
ACT	0.542	13	1.088	13	1.35	14	1.14	13	13.24	14
CAC	0.068	3	0.123	2	0.89	7	0.46	2	3.03	3
CYP	0.411	11	0.471	10	0.86	6	0.67	10	9.01	11
DNAJ	0.032	1	0.113	1	0.89	8	0.45	1	1.68	1
DREB	0.1	4	0.209	4	0.96	12	0.49	4	5.26	6
EF1	0.335	9	0.327	9	0.63	2	0.59	9	6.18	7
RAN	0.166	5	0.285	7	1.08	13	0.54	7	7.51	10

TIF1	0.446	12	0.51	12	0.95	11	0.7	12	11.74	13
TUB	0.376	10	0.51	11	0.94	10	0.69	11	10.49	12
UBC2	0.032	1	0.14	3	0.9	9	0.46	3	3	2
ABCC2	0.225	6	0.256	5	0.75	4	0.51	5	4.95	4
COPS3	0.645	14	1.212	14	0.47	1	1.26	14	7.24	9
CS	0.288	8	0.316	8	0.85	5	0.55	8	7.11	8
R3HDM2	0.263	7	0.273	6	0.68	3	0.53	6	5.24	5
**Osmotic_Leaf**										
ACT	1.226	12	1.498	11	3.1	13	1.8	12	11.98	14
CAC	0.808	6	0.885	8	1.89	4	1.35	9	6.45	8
CYP	0.858	7	1.995	14	1.76	3	1.29	7	5.45	6
DNAJ	0.274	1	0.664	5	2.27	6	1.15	1	2.55	2
DREB	1.352	13	0.707	6	1.23	2	1.95	13	8.14	10
EF1	1.059	10	0.526	3	3.19	14	1.68	10	10.88	12
RAN	0.274	1	1.786	13	2.27	7	1.22	5	3.31	3
TIF1	0.532	4	1.452	10	2.38	9	1.15	1	2.45	1
TUB	1.142	11	0.481	2	2.9	12	1.77	11	11.49	13
UBC2	0.707	5	0.655	4	1.97	5	1.26	6	5.69	7
ABCC2	0.945	9	0.438	1	2.74	11	1.33	8	9.19	11
COPS3	1.465	14	1.576	12	0.92	1	2.14	14	7.24	9
CS	0.325	3	0.71	7	2.3	8	1.21	4	4.68	5
R3HDM2	0.902	8	0.944	9	2.48	10	1.19	3	4.68	4
**Osmotic_Root**										
ACT	0.324	12	0.402	12	1.09	12	0.52	12	12	13
CAC	0.087	1	0.14	3	0.96	6	0.35	1	2.45	2
CYP	0.269	10	0.299	10	0.99	9	0.47	10	9.74	11

DNAJ	0.087	1	0.123	1	0.97	7	0.35	1	1.63	1
DREB	0.117	3	0.135	2	0.94	4	0.36	3	2.91	3
EF1	0.3	11	0.396	11	1.12	13	0.52	11	11.47	12
RAN	0.221	8	0.187	5	1.06	11	0.38	4	6.48	7
TIF1	0.356	13	0.472	13	1.13	14	0.6	13	13.24	14
TUB	0.238	9	0.187	6	1	10	0.39	6	7.54	10
UBC2	0.143	5	0.183	4	0.95	5	0.39	5	4.73	4
ABCC2	0.168	6	0.23	7	0.85	3	0.42	8	5.63	5
COPS3	0.496	14	1.31	14	0.35	1	1.34	14	7.24	9
CS	0.122	4	0.233	8	0.98	8	0.39	7	6.51	8
R3HDM2	0.197	7	0.246	9	0.84	2	0.44	9	5.8	6
**Salt_Leaf**										
ACT	0.904	13	1.625	13	2.45	14	1.75	13	13.24	14
CAC	0.142	1	0.538	5	1.13	6	0.85	4	3.31	3
CYP	0.353	7	0.325	2	1.18	7	0.82	1	3.15	2
DNAJ	0.142	1	0.54	6	1.11	5	0.84	3	3.08	1
DREB	0.309	6	0.598	9	0.85	1	0.91	8	4.56	5
EF1	0.759	12	0.947	12	2.03	13	1.22	12	12.24	13
RAN	0.243	4	0.664	10	0.94	2	0.9	7	4.86	7
TIF1	0.512	9	0.437	3	1.54	10	0.96	10	7.21	9
TUB	0.683	11	0.891	11	1.71	12	1.18	11	11.24	11
UBC2	0.201	3	0.597	8	0.97	4	0.87	6	4.9	8
ABCC2	0.413	8	0.291	1	1.37	9	0.83	2	3.46	4
COPS3	1.075	14	1.974	14	1.31	8	2.1	14	12.17	12
CS	0.276	5	0.553	7	0.95	3	0.86	5	4.79	6
R3HDM2	0.591	10	0.496	4	1.6	11	0.96	9	7.93	10

**Salt_Root**										
ACT	0.708	14	1.28	14	1.09	14	1.35	14	14	14
CAC	0.601	13	1.064	13	0.77	10	1.16	13	12.17	13
CYP	0.35	9	0.395	9	0.8	12	0.62	9	9.67	10
DNAJ	0.183	3	0.07	1	0.55	3	0.49	1	1.86	2
DREB	0.127	1	0.115	3	0.51	1	0.51	2	1.73	1
EF1	0.403	11	0.533	11	0.92	13	0.72	11	11.47	12
RAN	0.317	8	0.283	6	0.59	6	0.57	6	6.45	6
TIF1	0.371	10	0.439	10	0.78	11	0.64	10	10.24	11
TUB	0.237	5	0.096	2	0.67	9	0.51	2	3.66	4
UBC2	0.127	1	0.237	5	0.55	3	0.54	5	2.94	3
ABCC2	0.288	7	0.371	8	0.65	8	0.61	8	7.74	9
COPS3	0.507	12	0.973	12	0.52	2	1.09	12	7.67	8
CS	0.21	4	0.121	4	0.56	5	0.52	4	4.23	5
R3HDM2	0.273	6	0.332	7	0.64	7	0.59	7	6.74	7
**ABA_Leaf**										
ACT	0.3	10	0.293	8	0.31	4	0.42	9	7.33	9
CAC	0.17	3	0.123	2	0.35	9	0.33	2	3.22	2
CYP	0.274	9	0.191	4	0.32	5	0.37	5	5.73	7
DNAJ	0.205	5	0.302	9	0.46	12	0.39	8	8.11	10
DREB	0.226	7	0.406	11	0.48	14	0.47	11	10.44	12
EF1	0.357	12	0.451	12	0.33	7	0.54	12	10.49	13
RAN	0.217	6	0.383	10	0.47	13	0.45	10	9.4	11
TIF1	0.191	4	0.254	7	0.32	6	0.39	6	5.38	5
TUB	0.396	13	0.557	14	0.23	1	0.61	13	6.97	8
UBC2	0.144	1	0.216	5	0.36	10	0.35	4	3.76	3

ABCC2	0.254	8	0.134	3	0.34	8	0.35	3	4.9	4
COPS3	0.428	14	0.546	13	0.43	11	0.62	14	12.94	14
CS	0.144	1	0.014	1	0.29	3	0.32	1	1.32	1
R3HDM2	0.317	11	0.235	6	0.27	2	0.39	7	5.51	6
**ABA_Root**										
ACT	0.658	14	1	14	1.15	13	1.07	14	13.74	14
CAC	0.129	1	0.064	1	0.89	7	0.47	1	1.63	1
CYP	0.489	11	0.488	10	0.94	9	0.68	10	9.97	12
DNAJ	0.194	5	0.298	5	0.9	8	0.55	5	5.62	7
DREB	0.165	4	0.286	4	0.97	11	0.53	4	5.15	5
EF1	0.464	10	0.51	11	0.76	5	0.68	11	9.23	11
RAN	0.129	1	0.208	2	0.96	10	0.5	2	2.51	2
TIF1	0.517	12	0.589	12	1.19	14	0.72	12	12.47	13
TUB	0.416	8	0.413	8	0.61	3	0.61	7	6.05	8
UBC2	0.151	3	0.267	3	0.99	12	0.52	3	4.24	3
ABCC2	0.441	9	0.484	9	0.76	6	0.65	9	7.77	9
COPS3	0.59	13	0.98	13	0.5	1	1.05	13	8.14	10
CS	0.309	6	0.318	6	0.7	4	0.56	6	5.42	6
R3HDM2	0.376	7	0.392	7	0.5	1	0.62	8	4.45	4
**MeJA_Leaf**										
ACT	0.388	7	0.382	8	0.73	12	0.58	7	8.28	10
CAC	0.271	4	0.147	2	0.6	7	0.5	1	3.25	3
CYP	0.369	6	0.276	3	0.62	8	0.55	4	4.9	5
DNAJ	0.203	3	0.346	6	0.62	9	0.56	5	5.33	7
DREB	0.16	1	0.417	9	0.62	10	0.6	8	5.18	6
EF1	0.443	10	0.361	7	0.47	3	0.61	9	6.59	8

RAN	0.478	12	0.51	12	0.8	13	0.68	12	12.24	12
TIF1	0.46	11	0.487	11	0.53	5	0.67	11	9.03	11
TUB	0.509	13	0.597	13	0.65	11	0.75	13	12.47	13
UBC2	0.16	1	0.29	4	0.57	6	0.53	3	2.91	2
ABCC2	0.332	5	0.101	1	0.46	2	0.5	1	1.78	1
COPS3	0.67	14	1.592	14	0.87	14	1.64	14	14	14
CS	0.429	9	0.423	10	0.51	4	0.63	10	7.75	9
R3HDM2	0.412	8	0.32	5	0.43	1	0.58	6	3.94	4
**MeJA_Root**										
ACT	0.575	14	1.02	14	0.98	14	1.06	14	14	14
CAC	0.324	7	0.123	1	0.54	7	0.44	1	2.65	1
CYP	0.409	11	0.427	11	0.72	13	0.59	11	11.47	12
DNAJ	0.294	6	0.198	4	0.57	8	0.47	5	5.57	7
DREB	0.388	10	0.372	9	0.63	10	0.53	8	9.43	11
EF1	0.203	3	0.33	7	0.35	1	0.51	7	4.14	4
RAN	0.365	9	0.296	6	0.63	10	0.5	6	7.54	10
TIF1	0.43	12	0.485	12	0.68	12	0.61	12	12	13
TUB	0.252	5	0.139	2	0.45	4	0.44	1	3.31	2
UBC2	0.343	8	0.166	3	0.59	9	0.44	1	4.56	6
ABCC2	0.157	1	0.211	5	0.46	6	0.45	4	3.31	3
COPS3	0.494	13	0.86	13	0.35	1	0.91	13	6.85	9
CS	0.157	1	0.37	8	0.45	4	0.53	8	4.36	5
R3HDM2	0.226	4	0.396	10	0.43	3	0.56	10	5.89	8

Table S5 Stability of candidate reference genes under different conditions in *G. inflata*.

Candidate genes	geNorm		NormFinder		BestKeeper		ΔCt method		RefFinder method	
	Stability	Ranking	Stability	Ranking	Stability	Ranking	Stability	Ranking	Stability	Ranking
**Control_Leaf**										
ACT	0.958	14	2.821	14	2.89	14	2.84	14	14	14
CAC	0.573	11	0.762	12	0.4	2	0.92	11	7.34	9
CYP	0.244	3	0.288	4	0.54	6	0.77	7	4.74	4
DNAJ	0.607	12	0.899	13	0.46	3	1.02	13	8.83	11
DREB	0.381	5	0.294	5	0.7	12	0.74	5	6.22	7
EF1	0.644	13	0.541	9	0.97	13	0.95	12	11.62	13
RAN	0.283	4	0.149	3	0.46	4	0.68	1	2.63	3
TIF1	0.463	8	0.303	6	0.64	10	0.81	8	7.87	10
TUB	0.135	1	0.068	1	0.65	11	0.71	3	2.4	2
UBC2	0.439	7	0.456	8	0.51	5	0.75	6	6.4	8
ABCC2	0.543	10	0.736	11	0.63	8	0.91	10	9.97	12
COPS3	0.135	1	0.068	2	0.56	7	0.69	2	2.3	1
CS	0.411	6	0.342	7	0.63	9	0.73	4	6.05	6
R3HDM2	0.506	9	0.568	10	0.31	1	0.88	9	5.33	5
**Control_Root**										
ACT	0.612	11	0.829	12	0.57	1	0.96	12	6.31	7
CAC	0.228	1	0.269	2	0.94	9	0.62	2	2.45	2
CYP	0.765	14	0.862	14	1.25	12	0.99	14	13.47	14
DNAJ	0.53	9	0.722	10	0.81	6	0.86	10	8.57	11
DREB	0.431	5	0.454	8	0.87	8	0.7	8	7.11	8
EF1	0.68	12	0.822	11	1.42	14	0.95	11	11.94	12
RAN	0.483	7	0.42	7	1.09	11	0.69	6	7.54	9

TIF1	0.455	6	0.406	4	0.76	4	0.67	5	4.68	5
TUB	0.727	13	0.836	13	1.3	13	0.97	13	13	13
UBC2	0.399	4	0.408	5	0.79	5	0.65	4	4.47	4
ABCC2	0.365	3	0.517	9	0.74	2	0.73	9	4.7	6
COPS3	0.564	10	0.413	6	1.04	10	0.7	7	8.05	10
CS	0.228	1	0.112	1	0.83	7	0.58	1	1.63	1
R3HDM2	0.502	8	0.314	3	0.75	3	0.65	3	3.83	3
**Osmotic_Leaf**										
ACT	0.959	14	2.807	14	2.43	14	2.83	14	14	14
CAC	0.3	5	0.356	5	0.4	4	0.81	7	5.62	6
CYP	0.255	3	0.375	7	0.42	6	0.82	9	5.8	7
DNAJ	0.276	4	0.185	2	0.28	1	0.74	3	2.63	3
DREB	0.611	12	0.704	12	0.59	12	0.9	12	12	12
EF1	0.555	10	0.555	10	0.46	8	0.84	10	9.46	10
RAN	0.204	1	0.26	3	0.51	9	0.8	6	3.57	4
TIF1	0.493	8	0.415	8	0.55	11	0.81	7	8.38	9
TUB	0.204	2	0.092	1	0.4	4	0.73	1	1.68	1
UBC2	0.648	13	0.951	13	0.6	13	1.05	13	13	13
ABCC2	0.588	11	0.652	11	0.54	10	0.87	11	10.74	11
COPS3	0.441	7	0.363	6	0.28	1	0.73	1	2.55	2
CS	0.53	9	0.472	9	0.45	7	0.77	5	7.3	8
R3HDM2	0.393	6	0.29	4	0.34	3	0.74	3	4.12	5
**Osmotic_Root**										
ACT	0.629	14	1.226	14	0.64	8	1.27	14	12.54	13
CAC	0.377	8	0.343	9	0.62	7	0.57	8	7.97	8
CYP	0.394	9	0.342	8	0.75	12	0.56	7	8.82	10

DNAJ	0.436	11	0.507	11	0.68	10	0.66	11	10.74	11
DREB	0.249	4	0.371	10	0.7	11	0.58	10	8.14	9
EF1	0.407	10	0.338	7	0.58	4	0.57	9	7.09	7
RAN	0.459	12	0.558	12	0.77	13	0.69	12	12.24	12
TIF1	0.159	1	0.08	1	0.64	9	0.44	1	1.68	1
TUB	0.521	13	0.843	13	0.97	14	0.92	13	13.24	14
UBC2	0.193	3	0.221	4	0.54	3	0.5	4	3.46	4
ABCC2	0.323	6	0.171	3	0.44	2	0.49	3	3.22	3
COPS3	0.289	5	0.273	5	0.61	6	0.53	5	5.23	6
CS	0.353	7	0.308	6	0.36	1	0.56	6	3.98	5
R3HDM2	0.159	1	0.153	2	0.59	5	0.47	2	2.11	2
**Salt_Leaf**										
ACT	1.415	14	3.093	14	1.86	13	3.16	14	13.74	14
CAC	0.211	3	0.392	1	0.69	6	1.03	1	2.06	1
CYP	0.287	5	0.64	9	0.74	8	1.14	6	6.82	7
DNAJ	0.249	4	0.44	3	0.7	7	1.06	2	3.6	5
DREB	0.706	9	0.559	5	0.86	9	1.18	8	7.54	8
EF1	0.607	8	0.74	11	0.59	5	1.26	11	8.34	9
RAN	0.096	1	0.577	6	0.52	3	1.09	3	2.71	2
TIF1	1.124	13	2.784	13	2.82	14	2.85	13	13.24	13
TUB	0.096	1	0.581	7	0.55	4	1.09	4	3.25	4
UBC2	0.846	12	0.785	12	1.16	12	1.27	12	12	12
ABCC2	0.813	11	0.682	10	0.98	11	1.21	10	10.49	11
COPS3	0.459	6	0.397	2	0.43	1	1.11	5	2.78	3
CS	0.772	10	0.584	8	0.89	10	1.19	9	9.21	10
R3HDM2	0.54	7	0.533	4	0.47	2	1.16	7	4.45	6

**Salt_Root**									
ACT	1.187	13	2.679	13	2.35	13	2.85	13	13	13
CAC	0.368	4	0.396	4	0.4	3	1.01	1	2.83	2
CYP	0.553	8	0.182	3	0.61	9	1.04	3	5.05	6
DNAJ	0.446	6	0.441	5	0.25	1	1.08	5	3.5	3
DREB	0.882	12	1.823	12	1.3	12	2.05	12	12	12
EF1	0.402	5	0.644	8	0.4	3	1.1	7	5.38	8
RAN	0.258	3	0.631	7	0.42	5	1.1	8	5.38	9
TIF1	1.436	14	2.766	14	2.46	14	2.93	14	14	14
TUB	0.623	10	0.916	11	0.79	11	1.29	10	10.49	11
UBC2	0.708	11	0.621	6	0.72	10	1.33	11	9.23	10
ABCC2	0.517	7	0.091	1	0.34	2	1.03	2	2.74	1
COPS3	0.173	1	0.692	9	0.45	6	1.09	6	4.24	5
CS	0.578	9	0.091	1	0.49	7	1.07	4	3.98	4
R3HDM2	0.173	1	0.822	10	0.53	8	1.16	9	5.18	7
**ABA_Leaf**										
ACT	0.69	14	0.995	14	0.89	13	1.08	14	13.74	14
CAC	0.565	12	0.718	12	0.59	11	0.84	12	11.74	12
CYP	0.315	5	0.317	3	0.5	7	0.57	3	4.79	5
DNAJ	0.385	7	0.472	8	0.62	12	0.64	8	8.56	8
DREB	0.14	1	0.33	4	0.3	3	0.57	3	2.63	3
EF1	0.625	13	0.801	13	0.94	14	0.93	13	13.24	13
RAN	0.367	6	0.408	6	0.56	9	0.6	6	6.64	7
TIF1	0.491	10	0.53	11	0.55	8	0.73	10	9.92	11
TUB	0.412	8	0.508	9	0.58	10	0.68	9	8.97	9
UBC2	0.14	1	0.343	5	0.28	1	0.57	3	1.97	1

ABCC2	0.145	3	0.455	7	0.32	4	0.63	7	4.92	6
COPS3	0.437	9	0.164	1	0.35	5	0.56	2	3.08	4
CS	0.171	4	0.238	2	0.29	2	0.53	1	2	2
R3HDM2	0.529	11	0.518	10	0.43	6	0.73	10	9.01	10
**ABA_Root**										
ACT	0.943	14	2.551	14	2.45	14	2.59	14	14	14
CAC	0.377	5	0.111	1	0.69	4	0.67	1	2.24	1
CYP	0.614	12	0.486	7	0.43	1	0.87	10	5.38	5
DNAJ	0.488	8	0.312	4	0.91	12	0.76	4	6.26	7
DREB	0.197	1	0.687	11	0.74	9	0.87	11	5.74	6
EF1	0.669	13	0.71	12	1.13	13	1.05	13	12.74	13
RAN	0.458	7	0.584	9	0.69	4	0.82	8	6.7	11
TIF1	0.567	10	0.369	6	0.77	10	0.82	6	7.75	12
TUB	0.425	6	0.273	2	0.71	7	0.7	2	3.6	2
UBC2	0.22	3	0.637	10	0.73	8	0.82	7	6.4	9
ABCC2	0.197	1	0.725	13	0.77	10	0.89	12	6.44	10
COPS3	0.533	9	0.285	3	0.7	6	0.77	5	5.33	4
CS	0.266	4	0.356	5	0.64	3	0.71	3	3.66	3
R3HDM2	0.595	11	0.533	8	0.6	2	0.86	9	6.31	8
**MeJA_Leaf**										
ACT	0.861	14	3.006	14	2.83	14	3.02	14	14	14
CAC	0.123	3	0.345	7	0.53	10	0.63	4	5.69	6
CYP	0.166	4	0.375	9	0.53	10	0.63	4	6.31	8
DNAJ	0.1	1	0.311	4	0.55	12	0.62	3	3.46	4
DREB	0.428	11	0.33	5	0.48	7	0.78	10	7.88	9
EF1	0.501	13	0.384	10	0.49	8	0.83	13	10.78	13

RAN	0.1	1	0.334	6	0.51	9	0.61	1	2.71	1
TIF1	0.356	9	0.196	2	0.39	2	0.66	7	4.12	5
TUB	0.192	5	0.367	8	0.46	5	0.66	7	6.12	7
UBC2	0.244	6	0.248	3	0.4	4	0.61	1	3.46	3
ABCC2	0.299	7	0.665	13	0.59	13	0.79	11	10.68	12
COPS3	0.379	10	0.168	1	0.37	1	0.65	6	2.78	2
CS	0.329	8	0.559	12	0.47	6	0.75	9	8.49	11
R3HDM2	0.467	12	0.43	11	0.39	2	0.82	12	8.3	10
**MeJA_Root**										
ACT	0.558	12	0.626	10	0.52	3	0.8	12	8.11	9
CAC	0.367	6	0.339	7	0.73	9	0.58	5	6.9	7
CYP	0.622	13	0.856	13	0.7	7	0.97	13	11.14	12
DNAJ	0.387	7	0.483	9	0.87	12	0.66	9	9.08	10
DREB	0.257	3	0.281	3	0.5	1	0.57	3	2.45	2
EF1	0.511	11	0.639	11	0.88	13	0.77	10	11.2	13
RAN	0.339	5	0.328	5	0.75	10	0.57	3	5.23	5
TIF1	0.418	8	0.35	8	0.71	8	0.6	8	8	8
TUB	0.479	10	0.643	12	0.83	11	0.78	11	10.98	11
UBC2	0.233	1	0.155	1	0.54	4	0.51	1	1.41	1
ABCC2	0.233	1	0.318	4	0.55	5	0.58	5	3.16	4
COPS3	0.439	9	0.33	6	0.51	2	0.59	7	5.24	6
CS	0.306	4	0.198	2	0.61	6	0.54	2	3.13	3
R3HDM2	0.681	14	0.941	14	0.9	14	1.04	14	14	14

Table S6 Recommended reference genes for RT-qPCR analysis under different conditions in Leguminosae plants.

Species	Treatment	Candidate	Recommendation	Software	Reference
Glycyrrhiza uralensis F.	Osmotic stress-leaf	*ACT, CAC, CYP, DNAJ, DREB, EF1, RAN, TIF1, TUB, UBC2, ABCC2, COPS3, CS, R3HDM2*	*TIF1, DNAJ, RAN*	geNorm, NormFinder, BestKeeper, ΔCt, RefFinder	This study
Glycyrrhiza uralensis F.	Osmotic stress-root	*ACT, CAC, CYP, DNAJ, DREB, EF1, RAN, TIF1, TUB, UBC2, ABCC2, COPS3, CS, R3HDM2*	*DNAJ, CAC, DREB*	geNorm, NormFinder, BestKeeper, ΔCt, RefFinder	This study
Glycyrrhiza inflata B.	Osmotic stress-leaf	*ACT, CAC, CYP, DNAJ, DREB, EF1, RAN, TIF1, TUB, UBC2, ABCC2, COPS3, CS, R3HDM2*	*TUB, COPS3, DNAJ*	geNorm, NormFinder, BestKeeper, ΔCt, RefFinder	This study
Glycyrrhiza inflata B.	Osmotic stress-root	*ACT, CAC, CYP, DNAJ, DREB, EF1, RAN, TIF1, TUB, UBC2, ABCC2, COPS3, CS, R3HDM2*	*TIF1, R3HDM2, ABCC2*	geNorm, NormFinder, BestKeeper, ΔCt, RefFinder	This study
Ammopiptanthus mongolicus (Maxim. ex Kom.) Cheng f.	Drought stress	*eIF1, eIF3, Tub1, Abc1, EF1, Tub2, UBQ, Ubc1, Ubc2, Ubc4, eIF2, Ubc5, eIF4, EF2*	*eIF1, eIF3, UBQ*	geNorm, NormFinder	(Shi et al. 2012)
Arachis hypogaea L.	Drought stress	*CYP2, ELF1B, LEC, ADH3, ACT11, ATPsyn, G6PD, UBC1*	*CYP2, ELF1B, G6PD*	geNorm, NormFinder	(Reddy et al. 2013)
Arachis hypogaea L.	Osmotic stress	*YLS8, GAPDH, UKN2, ACTIN11, ACTIN7, ACTIN1, ADH3, ELF1B, G6PD, 60S, UBI1*	*ELF1B, YLS8, UBI1*	geNorm, NormFinder, BestKeeper	(He et al. 2017)
Cajanus cajan (Linn.) Millsp.	Drought stress	*IF4α, TUB6, HSP90, 18S rRNA, EF1α, UBQ10, GAPDH, 25S rRNA, ACT1, UBC*	*IF4α, TUB6, HSP90*	geNorm, NormFinder, BestKeeper	(Sinha et al. 2015)
Caragana intermedia	Osmotic stress leaves	*ACT7, TUA5, EF-1α, PP2A, SAND, TIP41, F-box, PEPKR1, UNK1, UNK2*	*TIP41, PP2A, SAND*	geNorm, NormFinder, BestKeeper	(Zhu et al. 2013)
Caragana intermedia	Osmotic stress roots	*ACT7, TUA5, EF-1α, PP2A, SAND, TIP41, F-box, PEPKR1, UNK1, UNK2*	*UNK1, UNK2, PP2A*	geNorm, NormFinder, BestKeeper	(Zhu et al. 2013)
Caragana korshinskii Kom	Drought stress	*GAPDH, EF-1α, SKIP5-1, TUA, TUB, SKIP1, UBQ, CAP, TUB3, SKIP5-2*	*GAPDH, EF-1α, SKIP5-1*	geNorm, NormFinder, BestKeeper	(Yang et al. 2014)

Cassia obtusifolia L.	Osmotic stress	*ACT2, UBQ1,β-TUB2, CYP1, EF1α2, UBQ2, ACT1, CYP2, α-TUB1, α-TUB2*	*ACT2, UBQ1, CYP1*	geNorm, NormFinder, BestKeeper	(Liu et al. 2015)
Cicer arietinum L.	Drought stress	*bHLH, CAC, UBQ, PUBQ, 18S rRNA, GAPDH, EF-1a, Helicase, TUB, ACT, PP2A, PPR*	*bHLH, CAC, UBQ*	geNorm	(Castro et al. 2012)
Cyamopsis tetragonoloba L.Taub	Drought stress	*ACT 7, TUB, 18S rRNA, CYP, ACT 11, EF-1α, TUA, UBQ 10, UBC 2, GAPDH*	*ACT7, TUB, ACT11*	geNorm, NormFinder, BestKeeper, ΔCt, RefFinder	(Jaiswal et al. 2019)
Glycine max (L.) Merr.	Drought stress	*NUDIX, NCL1,DNAJ,FYVE, CYP, TUB4, SKIP16, PEPKR1, TIP41, ELF1-B, TUA, B-actin, GAPDH, CYST, Golgin-84, NCL1, RNA-poly Mitovirus*	*NUDIX, NCL1, Golgin-84*	geNorm, NormFinder	(Marcolino-Gomes et al. 2015)
Glycine max (L.) Merr.	Drought stress	*TUB4, TUA5, EF1A, CYP, EF1B, TUA4, ACT2/7, ACT11, UKN2*	*TUB4, TUA5, EF1A*	geNorm, NormFinder	(Ma et al. 2013)
Glycine max (L.) Merr.	Osmotic stress	*60S, ABC, Act27, Act11, CDPK, CYP2, ELF1a, ELF1b, Fbox, IDE, SUBI2, TUBα, TUBβ*	*Fbox, 60S, ELF1b*	geNorm	(Le et al. 2012)
Glycine max (L.) Merr.	Osmotic stress root	*60S, ABC, Act27, Act11, CDPK, CYP2, ELF1a, ELF1b, Fbox, IDE, SUBI2, TUBα, TUBβ*	*Fbox, ABC, Act11*	geNorm	(Le et al. 2012)
Glycine max (L.) Merr.	Osmotic stress shoot	*60S, ABC, Act27, Act11, CDPK, CYP2, ELF1a, ELF1b, Fbox, IDE, SUBI2, TUBα, TUBβ*	*Fbox, 60S, Act11*	geNorm	(Le et al. 2012)
Glycyrrhiza glabra L.	Drought stress leaf	*UBC2, TUB, ACT, FBP, EF1, TIF1, GAPDH, HIS3*	*UBC2, TUB, ACT*	geNorm, NormFinder	(Maroufi 2016)
Glycyrrhiza glabra L.	Drought stress root	*UBC2, TUB, ACT, FBP, EF1, TIF1, GAPDH, HIS4*	*UBC2, TUB, ACT*	geNorm, NormFinder	(Maroufi 2016)
Oxytropis ochrocephala Bunge	Drought stress	*HIS, ACT101,TUA,ACT7, FBA, TUB, ACTB, GAPDH2, MET, ACT11, GAPDH1, 18S*	*HIS, ACT101, TUB*	geNorm, NormFinder, BestKeeper	(Zhuang et al. 2015)
Robinia pseudoacacia L.	Osmotic stress	*helicase, EF1α,18s rRNA, ACT, GAPDH, βTUB, UBQ, PP2A, CAC, SAMDC, CYP, SAND*	*ACT, GAPDH, helicase*	geNorm, NormFinder, BestKeeper	(Wang et al. 2018)

Trifolium repens L.	Drought stress	*EF1a, ACT11, UBQ, CYP, EF1b, G6PD, TUA, UBQ10*	*EF1a, ACT11, UBQ*	ΔCt, NormFinder, single-factor ANOVA	(Narancio et al. 2018)
Vigna angularis (Willd.) Ohwi et Ohashi	Drought stress	*Fbox, PTB,GAPDH,ACT, ZMPP, EF, PP2A, UBC, UBN*	*Fbox, PTB, EF*	geNorm, NormFinder, BestKeeper	(Chi et al. 2016)
Vigna mungo L.	Drought stress	*TUB, 18S,CYP,ACT, EF1A, 18S, H2A, GAPDH, CYP, TUB*	*TUB, 18S, ACT*	geNorm, NormFinder	(Kundu et al. 2013)
Vigna unguiculata L. Walp	Drought stress	*VuYls8, VuPolyP,VuUbq10,VuPp2A, VuUbq28, VuEF1-Alpha, VuAct2, VuGapdh, VusHsp17.7, VuNced1*	*PolyP, Pp2A, Ubq28*	geNorm, NormFinder	(Da Silva et al. 2015)
Vigna unguiculata L. Walp	Drought stress leaf	*VuPolyP, VuPp2A, VuUbq28,VuAct2,VuYls8, VuUbq10, VuEF1-Alpha, VuGapdh, VusHsp17.7, VuNced1*	*PolyP, Pp2A, Ubq28*	geNorm, NormFinder	(Da Silva et al. 2015)
Vigna unguiculata L. Walp	Drought stress nodule	*VuPp2A, VuYls8, VuUbq28,VuUbq10,VuEF1-Alpha, VuPolyP, VuAct2, VuGapdh, VusHsp17.7, VuNced1*	*PolyP, Pp2A, Ubq28*	geNorm, NormFinder	(Da Silva et al. 2015)
Glycyrrhiza uralensis F.	Salt stress-leaf	*ACT, CAC, CYP, DNAJ, DREB, EF1, RAN, TIF1, TUB, UBC2, ABCC2, COPS3, CS, R3HDM2*	*DNAJ, CYP, CAC*	geNorm, NormFinder, BestKeeper, ΔCt, RefFinder	This study
Glycyrrhiza uralensis F.	Salt stress-root	*ACT, CAC, CYP, DNAJ, DREB, EF1, RAN, TIF1, TUB, UBC2, ABCC2, COPS3, CS, R3HDM2*	*DREB, DNAJ, UBC2*	geNorm, NormFinder, BestKeeper, ΔCt, RefFinder	This study
Glycyrrhiza inflata B.	Salt stress-leaf	*ACT, CAC, CYP, DNAJ, DREB, EF1, RAN, TIF1, TUB, UBC2, ABCC2, COPS3, CS, R3HDM2*	*CAC, RAN, COPS3*	geNorm, NormFinder, BestKeeper, ΔCt, RefFinder	This study
Glycyrrhiza inflata B.	Salt stress-root	*ACT, CAC, CYP, DNAJ, DREB, EF1, RAN, TIF1, TUB, UBC2, ABCC2, COPS3, CS, R3HDM2*	*ABCC2, CAC, DNAJ*	geNorm, NormFinder, BestKeeper, ΔCt, RefFinder	This study

Ammopiptanthus mongolicus (Maxim. ex Kom.) Cheng f.	Salt stress	*eIF1, eIF3,Tub1, Abc1, EF1,Tub2, UBQ, Ubc1, Ubc2, Ubc4, eIF2, Ubc5, eIF4, EF2*	*eIF1, eIF3, Ubc2*	geNorm, NormFinder	(Shi et al. 2012)
Arachis hypogaea L.	Salt stress	*CYP2, ELF2B, LEC, ADH3, ACT11, ATPsyn, G6PD, UBC1*	*CYP2, ELF1B, G6PD*	geNorm, NormFinder	(Reddy et al. 2013)
Arachis hypogaea L.	Salt stress	*YLS8, GAPDH, UKN2, ACTIN11, ACTIN7, ACTIN1, ADH3, ELF1B, G6PD, 60S, UBI1*	*YLS8, GAPDH, ADH3*	geNorm, NormFinder, BestKeeper	(He et al. 2017)
Arachis hypogaea L.	Salt stress leaves	*TUA5, UKN2, TUB4, PEPKR1, UKN1, ACT2/7, ACT11, EF1b, CYP, UBQ10, TIP41, HDC, SKIP16, MTP*	*TUA5, UKN2, UKN1*	geNorm, NormFinder	(Chi et al. 2012)
Arachis hypogaea L.	Salt stress roots	*TUA5, UKN2, TUB4, PEPKR1, UKN1, ACT2/7, ACT11, EF1b, CYP, UBQ10, TIP41, HDC, SKIP16, MTP*	*HDC, UKN1, SKIP16*	geNorm, NormFinder	(Chi et al. 2012)
Cajanus cajan (Linn.) Millsp.	Salt stress	*GAPDH, UBC, HSP90, UBQ10, EF1α, 18SrRNA, 25SrRNA, TUB6, ACT1, IF4α*	*GAPDH, UBC, HSP90*	geNorm, NormFinder	(Sinha et al. 2015)
Caragana intermedia	Salt stress leaves	*ACT7, TUA5, EF-1α, PP2A, SAND, TIP41, F-box, PEPKR1, UNK1, UNK2*	*UNK2, SAND, EF-1α*	geNorm, NormFinder, BestKeeper	(Zhu et al. 2013)
Caragana intermedia	Salt stress roots	*ACT7, TUA5, EF-1α, PP2A, SAND, TIP41, F-box, PEPKR1, UNK1, UNK2*	*UNK2, SAND, TIP41*	geNorm, NormFinder, BestKeeper	(Zhu et al. 2013)
Caragana korshinskii Kom	Salt stress	*GAPDH, EF-1α, SKIP5-1,TUA, TUB, SKIP1,UBQ, CAP, TUB3, SKIP5-2*	*GAPDH, EF-1α, SKIP5-1*	geNorm, NormFinder, BestKeeper	(Yang et al. 2014)
Cassia obtusifolia L.	Salt stress	*ACT2, UBQ1,β-TUB2, CYP1, EF1α2, UBQ2, ACT1, CYP2, α-TUB1, α-TUB2*	*EF1α2, UBQ1, CYP1*	geNorm, NormFinder, BestKeeper	(Liu et al. 2015)
Cicer arietinum L.	Salt stress	*bHLH, CAC, UBQ, PUBQ, 18S rRNA, GAPDH, EF-1a, Helicase, TUB, ACT, PP2A, PPR*	*TUB, PP2A, EF1a*	geNorm	(Castro et al. 2012)

Cyamopsis tetragonoloba L.Taub	Salt stress	*ACT 7, TUB, 18S rRNA, CYP, ACT 11, EF-1α, TUA, UBQ 10, UBC 2, GAPDH*	*GAPDH, EF-1α, ACT 7*	geNorm, NormFinder, BestKeeper, ΔCt, RefFinder	(Jaiswal et al. 2019)
Glycine max (L.) Merr.	Salt stress	*EF1A, ACT11, Act2/7, TUA5, CYP, EF1B, TUA4, TUB4, UKN2*	*EF1A, ACT11, EF1B*	geNorm, NormFinder	(Ma et al. 2013)
Glycine max (L.) Merr.	Salt stress	*60S, ABC, Act27, Act11, CDPK, CYP2, ELF1a, ELF1b, Fbox, IDE, SUBI2, TUBα, TUBβ*	*60s, Fbox, ABC*	geNorm	(Le et al. 2012)
Glycine max (L.) Merr.	Salt stress root	*60S, ABC, Act27, Act11, CDPK, CYP2, ELF1a, ELF1b, Fbox, IDE, SUBI2, TUBα, TUBβ*	*ELF1b, IDE, ABC*	geNorm	(Le et al. 2012)
Glycine max (L.) Merr.	Salt stress shoot	*60S, ABC, Act27, Act11, CDPK, CYP2, ELF1a, ELF1b, Fbox, IDE, SUBI2, TUBα, TUBβ*	*Fbox, ELF1b, 60s*	geNorm	(Le et al. 2012)
Medicago sativa L.	Salt stress	*Msc27, ACT2, 18S rRNA,GAPDH, TUB, β -Actin,ELF-1α, UBC2*	*ELF-1α, Msc27, 18S rRNA*	geNorm, BestKeeper	(Wang et al. 2015)
Oxytropis ochrocephala Bunge	Salt stress	*HIS, ACT101,TUA,ACT7, FBA, TUB, ACTB, GAPDH2, MET, ACT11, GAPDH1, 18S*	*GAPDH2, HIS, ACT101*	geNorm, NormFinder, BestKeeper	(Zhuang et al. 2015)
Pisum sativum L.	Salt stress	*PP2A, GH720808, TUB, GH720838,18S rRNA,GH720843, Helicase, GAPDH, α-Tubuliin, Actin, EF-1α*	*PP2A, TUB, GH720838*	geNorm, qBase	(Die et al. 2010)
Robinia pseudoacacia L.	Salt stress	*helicase, EF1α,18s rRNA, ACT, GAPDH, βTUB, UBQ, PP2A, CAC, SAMDC, CYP, SAND*	*helicase, ACT, GAPDH*	geNorm, NormFinder, BestKeeper	(Wang et al. 2018)
Vigna angularis (Willd.) Ohwi et Ohashi	Salt stress	*Fbox, UBC, EF, ACT, ZMPP, GAPDH, PP2A, UBN, PTB*	*Fbox, UBC, PP2A*	geNorm, NormFinder, BestKeeper	(Chi et al. 2016)
Vigna mungo L.	Salt stress	*H2A, EF1A,18S,ACT, EF1A, 18S, H2A, GAPDH, CYP, TUB*	*H2A, EF1A, ACT*	geNorm, NormFinder	(Kundu et al. 2013)

Vigna unguiculata L. Walp	Salt stress	*UBC2, FBOX, UNK, EF1-α, β-TUB, Fbox, UE21D*	*UBC2, FBOX, UNK*	geNorm, NormFinder, BestKeeper, ΔCt	(Barbosa Amorim et al. 2018)
Glycyrrhiza uralensis F.	ABA treatment-leaf	*ACT, CAC, CYP, DNAJ, DREB, EF1, RAN, TIF1, TUB, UBC2, ABCC2, COPS3, CS, R3HDM2*	*CS, CAC, UBC2*	geNorm, NormFinder, BestKeeper, ΔCt, RefFinder	This study
Glycyrrhiza uralensis F.	ABA treatment-root	*ACT, CAC, CYP, DNAJ, DREB, EF1, RAN, TIF1, TUB, UBC2, ABCC2, COPS3, CS, R3HDM2*	*CAC, RAN, UBC2*	geNorm, NormFinder, BestKeeper, ΔCt, RefFinder	This study
Glycyrrhiza inflata B.	ABA treatment-leaf	*ACT, CAC, CYP, DNAJ, DREB, EF1, RAN, TIF1, TUB, UBC2, ABCC2, COPS3, CS, R3HDM2*	*UBC2, CS, DREB*	geNorm, NormFinder, BestKeeper, ΔCt, RefFinder	This study
Glycyrrhiza inflata B.	ABA treatment-root	*ACT, CAC, CYP, DNAJ, DREB, EF1, RAN, TIF1, TUB, UBC2, ABCC2, COPS3, CS, R3HDM2*	*CAC, TUB, CS*	geNorm, NormFinder, BestKeeper, ΔCt, RefFinder	This study
Caragana korshinskii Kom	ABA treatment	*GAPDH, EF-1α, SKIP5-1,TUA, TUB, SKIP1,UBQ, CAP, TUB3, SKIP5-2*	*GAPDH, EF-1α, SKIP5-1*	geNorm, NormFinder, BestKeeper	(Yang et al. 2014)
Cassia obtusifolia L.	ABA treatment	*ACT2, UBQ1, β-TUB2, CYP1, EF1α2, UBQ2, ACT1, CYP2, α-TUB1, α-TUB2*	*EF1α2, β-TUB2, CYP1*	geNorm, NormFinder, BestKeeper	(Liu et al. 2015)
Glycine max (L.) Merr.	ABA treatment	*60S, ABC, Act27, Act11, CDPK, CYP2, ELF1a, ELF1b, Fbox, IDE, SUBI2, TUBα, TUBβ*	*60s, ELF1b, ABC*	geNorm	(Le et al. 2012)
Glycine max (L.) Merr.	ABA treatment root	*60S, ABC, Act27, Act11, CDPK, CYP2, ELF1a, ELF1b, Fbox, IDE, SUBI2, TUBα, TUBβ*	*60s, ELF1b, ABC*	geNorm	(Le et al. 2012)

Glycine max (L.) Merr.	ABA treatment shoot	*60S, ABC, Act27, Act11, CDPK, CYP2, ELF1a, ELF1b, Fbox, IDE, SUBI2, TUBα, TUBβ*	*ELF1b, Fbox, ABC*	geNorm	(Le et al. 2012)
Medicago sativa L.	ABA treatment	*Msc27, ACT2, 18S rRNA,GAPDH, TUB, β -Actin,ELF-1α, UBC2*	*Msc27, Actin2, 18S rRNA*	geNorm, BestKeeper	(Wang et al. 2015)
Robinia pseudoacacia L.	ABA treatment	*ACT, GAPDH,CAC,βTUB, UBQ, EF1a, helicase, PP2A, 18s rRNA, SAMDC, CYP*	*ACT, GAPDH, helicase*	geNorm, NormFinder, BestKeeper	(Wang et al. 2018)
Glycyrrhiza uralensis F.	MeJA treatment-leaf	*ACT, CAC, CYP, DNAJ, DREB, EF1, RAN, TIF1, TUB, UBC2, ABCC2, COPS3, CS, R3HDM2*	*ABCC2, UBC2, CAC*	geNorm, NormFinder, BestKeeper, ΔCt, RefFinder	This study
Glycyrrhiza uralensis F.	MeJA treatment-root	*ACT, CAC, CYP, DNAJ, DREB, EF1, RAN, TIF1, TUB, UBC2, ABCC2, COPS3, CS, R3HDM2*	*CAC, TUB, ABCC2*	geNorm, NormFinder, BestKeeper, ΔCt, RefFinder	This study
Glycyrrhiza inflata B.	MeJA treatment-leaf	*ACT, CAC, CYP, DNAJ, DREB, EF1, RAN, TIF1, TUB, UBC2, ABCC2, COPS3, CS, R3HDM2*	*RAN, COPS3, UBC2*	geNorm, NormFinder, BestKeeper, ΔCt, RefFinder	This study
Glycyrrhiza inflata B.	MeJA treatment-root	*ACT, CAC, CYP, DNAJ, DREB, EF1, RAN, TIF1, TUB, UBC2, ABCC2, COPS3, CS, R3HDM2*	*UBC2, DREB, CS*	geNorm, NormFinder, BestKeeper, ΔCt, RefFinder	This study
Glycine max (L.) Merr.	MeJA treatment	*EF1A 1a1, EF1A 2b,ACT11,EF1A 2a, EF1B, UKN1, ACT, SKIP16*	*EF1A 2a, EF1A 1a1, EF1A 2b*	geNorm, NormFinder, BestKeeper, RefFinder	(Costa et al. 2016)
